# Predicting genotype-specific gene regulatory networks

**DOI:** 10.1101/gr.275107.120

**Published:** 2022-03

**Authors:** Deborah Weighill, Marouen Ben Guebila, Kimberly Glass, John Quackenbush, John Platig

**Affiliations:** 1 Harvard T.H. Chan School of Public Health, Boston, Massachusetts 02115, USA;; 2 Channing Division of Network Medicine, Brigham and Women's Hospital, Boston, Massachusetts 02115, USA;; 3 Harvard Medical School, Boston, Massachusetts 02115, USA

## Abstract

Understanding how each person's unique genotype influences their individual patterns of gene regulation has the potential to improve our understanding of human health and development, and to refine genotype-specific disease risk assessments and treatments. However, the effects of genetic variants are not typically considered when constructing gene regulatory networks, despite the fact that many disease-associated genetic variants are thought to have regulatory effects, including the disruption of transcription factor (TF) binding. We developed EGRET (Estimating the Genetic Regulatory Effect on TFs), which infers a genotype-specific gene regulatory network for each individual in a study population. EGRET begins by constructing a genotype-informed TF-gene prior network derived using TF motif predictions, expression quantitative trait locus (eQTL) data, individual genotypes, and the predicted effects of genetic variants on TF binding. It then uses a technique known as message passing to integrate this prior network with gene expression and TF protein–protein interaction data to produce a refined, genotype-specific regulatory network. We used EGRET to infer gene regulatory networks for two blood-derived cell lines and identified genotype-associated, cell line–specific regulatory differences that we subsequently validated using allele-specific expression, chromatin accessibility QTLs, and differential ChIP-seq TF binding. We also inferred EGRET networks for three cell types from each of 119 individuals and identified cell type–specific regulatory differences associated with diseases related to those cell types. EGRET is, to our knowledge, the first method that infers networks reflective of individual genetic variation in a way that provides insight into the genetic regulatory associations driving complex phenotypes.

The genetic architecture of complex human traits, especially disease traits, has been widely investigated in genome-wide association studies (GWAS), leading to the identification of genetic variants correlated with disease traits (Buniello et al. 2019). As the sample sizes of these studies have grown, it has become increasingly clear that many diseases and the traits that contribute to them are influenced by a very large number of variants, each with a relatively small effect size (Loh et al. 2015; Hall et al. 2016; Boyle et al. 2017). Furthermore, these GWAS variants are predominately located in noncoding regions of the genome and likely influence the regulation of gene expression (Finucane et al. 2015; Zhu et al. 2016; Gusev et al. 2018).

Expression quantitative trait locus (eQTL) analyses, which quantify associations between genetic variation and gene expression (Westra et al. 2013; The GTEx Consortium 2020), have helped to refine disease-associated genetic signals (Schaid et al. 2018) and enabled the identification of tissue-specific genetic effects (Barbeira et al. 2021). Whereas eQTL studies have helped to identify the genes that are potentially affected by GWAS variants, in isolation, they are unable to determine the regulatory mechanisms that mediate these interactions. There is, however, recent evidence that disease-associated genetic variants impact gene regulation, and thus expression, by disrupting transcription factor (TF) binding (Kalita et al. 2018; Vierstra et al. 2020). In addition, work by van de Geijn and colleagues has shown that genetic variation in transcription factor binding sites and their flanking regions explains a substantial amount of the heritability of many diseases and complex traits (van de Geijn et al. 2020).

Taken together, this suggests that a major consequence of trait-associated genetic variation is the alteration of the gene regulatory networks (GRNs) that are formed by the regulatory relationships between TFs and their target genes. Given that there are approximately 1600 TFs in the human genome (Lambert et al. 2018), direct observation of genome-wide TF regulatory networks is not currently experimentally feasible, particularly as patterns of regulation can change between biological states. An alternative is to use inference methods to estimate the TF-gene edges that form GRNs (Margolin et al. 2006; Faith et al. 2007; Glass et al. 2013). These methods have been used to identify regulatory changes that distinguish phenotypes (Basso et al. 2005; Neph et al. 2012; Sonawane et al. 2017), but they do not account for individual genetic differences and so also fail to provide a mechanistic link between genotype and phenotype.

Here, we aim to fill this gap by developing EGRET (Estimating the Genetic Regulatory Effect on TFs), a multi-omic network inference method that incorporates genotypes, eQTL information, and TF binding predictions with gene coexpression and protein–protein interactions to estimate individual-specific GRNs. We endeavor to demonstrate EGRET's utility through two analyses using publicly available omics resources. In our first application, we compared EGRET networks from two cell lines where a wealth of validation data are available. The second application represents a likely use case, where we built and compared EGRET networks in three different cell types for a population of individuals. Our aim in selecting these applications was to demonstrate EGRET's performance on real-world multi-omic data and provide examples of how to extract biological insights from these networks.

## Results

### The EGRET algorithm

**Figure 1.**
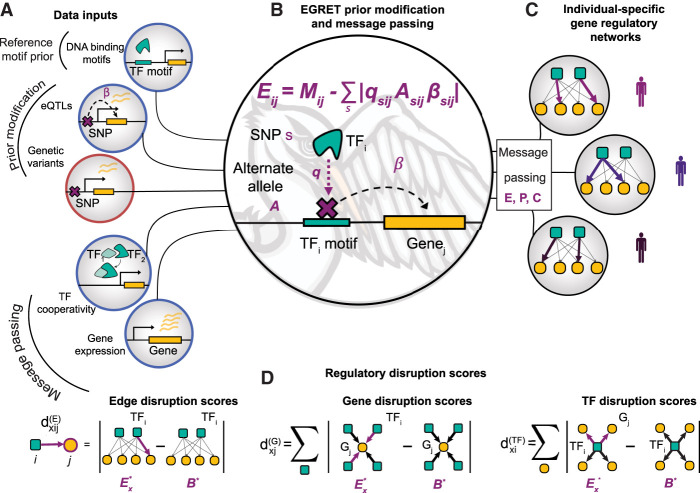
EGRET integrates multiple data types to construct individual-specific GRNs. (*A*) EGRET takes as input several data types (population-level inputs circled in blue, individual-specific inputs circled in orange) to construct individual-specific GRNs: an initial estimate of the binding locations of TFs in the form of a reference motif prior (*M*_*ij*_), the beta values of eQTL associations between “eSNPs” and “eGenes” (*β*), the genetic variants (*s*) harbored by the individual in question, PPI data as an estimate of TF–TF cooperativity (*P*), and gene expression to estimate a gene coexpression matrix (*C*). (*B*) An individual's genetic variants are used to modify the reference motif prior to produce an individual-specific EGRET prior (*E*) by penalizing motif-gene connections in which that individual carries a variant allele (*A*) in the relevant promoter-region motif such that the variant is an eQTL for the adjacent gene (*β*) and the variant is predicted by QBiC to affect TF binding at that location (*q*). (*C*) Message passing is used to integrate the coexpression (*C*) and PPI (*P*) networks with the EGRET prior (*E*) resulting in a final, unique GRN per individual (E*). (*D*) Regulatory disruption scores can be calculated to quantify the extent to which an edge or node in the network is disrupted by variants. Edge disruption scores are calculated by subtracting a genotype-agnostic baseline network (B*) from the individual's EGRET network and taking the absolute value. TF or gene disruption scores are calculated taking the sum of the edge disruption scores around the TF or gene in question.

EGRET uses several sources of information to capture the impact of genetic variants on TF-to-gene regulatory relationships and construct individual-specific GRNs (see Methods; Table 1; Fig. 1A; Supplemental Table S1; Supplemental Fig. S1). There are two main steps to the method: (1) generation of an individual-specific “EGRET prior” *E*, which serves as an initial estimate of the regulatory network; and (2) integration of *E* with gene-gene coexpression and TF–TF protein–protein interaction data using message passing. Once the integration is completed, EGRET returns an edge weight for every TF-gene pair that reflects the confidence of a regulatory relationship between the TF and its target gene, taking into account potential disruption by genetic variants.

**Table 1.**
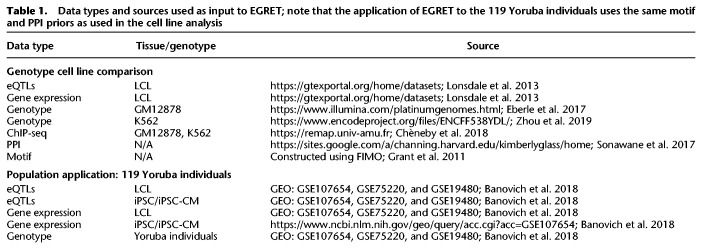
Data types and sources used as input to EGRET; note that the application of EGRET to the 119 Yoruba individuals uses the same motif and PPI priors as used in the cell line analysis

### Constructing the EGRET prior *E*

To construct the EGRET network prior *E*, EGRET requires four data inputs. The first is a TF-to-gene reference motif prior network, *M*, that estimates which TFs bind to promoter regions to regulate target gene expression. This can be derived, for example, from motif scans of a reference genome (Supplemental Note S1; Grant et al. 2011). Second, EGRET requires eQTL data either from the study population or from a public database from the cell type of interest. Third, EGRET uses genotype information in the form of genetic variants of the individual(s) for which GRNs are being constructed, and fourth, EGRET uses predictions of the effect of these genetic variants on TF binding (Supplemental Note S2) to modify the reference motif prior *M*.

These data are combined as follows: For each input genotype, EGRET selects SNPs (*A* in Fig. 1B) that (1) are within motif-based TF binding sites in the promoter regions of genes and (2) have a statistically significant eQTL association (*β* in Fig. 1B; Supplemental Fig. S2A,B) with the expression of the adjacent gene. EGRET then uses QBiC (Martin et al. 2019) to identify SNPs within TF motifs that significantly affect TF binding (significant negative QBiC values, *q*, in Fig. 1B) (for details, see Supplemental Note S2), thus selecting genetic variants in each individual that are predicted to affect both gene expression and TF binding (Supplemental Fig. S2C,D; Supplemental Table S2). The effect of a SNP *s* on TF *i*’s regulation of gene *j* is then defined as the product $\left|q_{s_{ij}}A_{s_{ij}}{\text{β}}_{s_{ij}}\right|$. The absolute value reflects the fact that EGRET edge weights estimate the strength of connection between a TF and its target gene but do not distinguish between disruption of activators and disruption of repressors. Modifier weights to the reference motif prior, *M*, are calculated by including these effects per TF-gene pair, allowing for the fact that a gene might have more than one variant in its promoter region affecting the binding of a particular TF. The EGRET prior network *E* is constructed by subtracting the modifier from the reference motif prior

$$E_{ij}=M_{ij}-\underset{s}{\sum }|q_{s_{ij}}A_{s_{ij}}{\text{β}}_{s_{ij}}|,$$

thus penalizing the reference motif prior when the individual in question contains a genetic variant with sufficient evidence to suggest it may alter gene regulation (Fig. 1B; Supplemental Fig. S3).

### Data integration using message passing

After constructing *E*, EGRET integrates this with gene expression and protein–protein interaction data (Supplemental Note S4) using a message passing framework (Supplemental Note S5; Glass et al. 2013). This framework takes as its input three networks: (1) an initial estimate of the TF-gene regulatory network (in our case, *E*); (2) a gene coexpression network, *C*, representing potential coregulatory relationships between genes calculated as the Pearson correlation coefficient between gene expression profiles; and (3) a PPI network, *P*, representing which TFs may physically interact to form TF–TF protein complexes. Thresholds are not applied to these network edge weights. At each iteration, the similarity between these networks is calculated. The edge weights in all three networks are then incrementally updated to reflect information from the other networks using the *availability* and *responsibility* functions. For each TF-gene pair *ij* in *E*, the *availability* of the edge represents the similarity between the target genes of TF *i* in *E* and the set of genes with which gene *j* is coexpressed in *C*. For the same edge in *E*, the *responsibility* represents the similarity between the set of TFs that target gene *j* in *E* and the interaction partners of TF *i* in *P*. Edge weights in *E* are then updated with a small fraction (*α* = 0.1) of the average of the *responsibility* and *availability*. Each edge in *C* and *P* is also updated with a small fraction of the overlap of the neighbors of pairs of genes/TFs in *E*, respectively. For example, the edge *kl* in *P* will be updated with the similarity between the set of target genes of TFs *k* and *l* in *E*. These updates of *E*, and then *C* and *P*, are repeated until convergence is achieved, defined as a Hamming distance between networks <10^−3^. Upon convergence, the primary output is an individual-specific, complete, bipartite GRN (E*) that captures genotype-specific regulatory effects. EGRET repeats this process separately using genotype information for each individual, producing a collection of individual-specific genotype-informed GRNs (Fig. 1C).

In addition, a “genotype agnostic” baseline GRN (B*) can be constructed using the TF-gene motif scan *M*, instead of the EGRET prior, *E*. This is achieved by applying the message passing framework described above to inputs *M*, *P*, and *C*, as opposed to *E*, *P*, and *C*. As *M* is derived from the reference genome, no genotype-specific information is included, providing a baseline GRN for comparison with the genotype-specific GRNs.

These networks, E*, can then be examined to identify features that are unique to specific genotypes, are associated with particular phenotypic states, or both. It is important to note that EGRET GRNs E* are complete graphs, meaning that an edge exists between all TFs and genes considered. However, it is the edge weights that indicate the strength of the relationship between the respective TFs and genes, with a higher weight indicating a higher likelihood of a regulatory relationship.

It is also worth noting that the data for *M*, *P*, and *C*, as well as the eQTLs can be obtained from publicly available resources. Thus, one can construct an EGRET network for a given cell type in an individual of interest simply by providing the genotype information for that individual and relying on publicly available data (for example, databases such as the Genotype-Tissue Expression Project [GTEx] [Lonsdale et al. 2013]) for the remaining model inputs. The EGRET algorithm is sufficiently efficient to be applied to large populations of individuals (Supplemental Note S6; Supplemental Table S3).

### Regulatory disruption scores

EGRET inferred edge weights can be used to quantitatively estimate the predicted regulatory effects produced by SNPs on a given gene, TF, or TF-gene relationship (Supplemental Table S4). A higher edge weight between a TF *i* and a gene *j* is interpreted as a higher confidence that the TF binds the promoter of and regulates the expression of gene *j*. To assess the effects of SNPs on gene regulation, we define and calculate three different regulatory disruption scores for nodes and edges in a given genotype *x* (Fig. 1D). The *edge disruption score* $d_{x_{ij}}^{\left(E\right)}$ quantifies the extent to which a TF-gene regulatory relationship is disrupted by genetic variants. The *gene disruption score* $d_{x_{j}}^{\left(G\right)}$ assesses the extent to which a gene has disrupted regulation due to genetic variants in its promoter region. The *TF disruption score* $d_{x_{i}}^{\left(TF\right)}$ represents the cumulative impact of *cis*-acting variants that disrupt a TF's regulation of its target genes. A TF with a high disruption score would suggest that many of its TF → gene edges have been disrupted by genetic variants, and thus the overall regulatory influence of the TF is diminished. These scores are defined per edge/node in each genotype-specific EGRET network by comparing it with a baseline network constructed using no genotype information and applying message passing to *M*, *P*, and *C* (instead of *E*, *P*, and *C*). For example, the edge disruption score is defined as

$$d_{x_{ij}}^{\left(E\right)}=|E_{x_{ij}}^{*}-B_{ij}^{*}|,$$

where $E_{x_{ij}}^{*}$ denotes the weight of edge *ij* in the EGRET network for individual *x* and $B_{ij}^{*}$ is the edge weight for edge *ij* in the baseline network predicted without using genotype information. This score quantifies the extent to which edges are disrupted by variants in a given individual-specific network (E*) compared with a baseline genotype-agnostic regulatory network (B*).

Similarly, TF disruption scores $d_{x_{i}}^{\left(TF\right)}$ and gene disruption scores $d_{x_{j}}^{\left(G\right)}$ are calculated by taking the sum of edge disruption scores around the specific TF or gene in question

$$d_{x_{i}}^{\left(TF\right)}=\underset{j}{\sum }|E_{x_{ij}}^{*}-B_{ij}^{*}|$$

$$d_{x_{j}}^{\left(G\right)}=\underset{i}{\sum }|E_{x_{ij}}^{*}-B_{ij}^{*}|.$$

It is worth noting that disruption scores are all greater than or equal to zero and that a higher edge disruption score corresponds to a larger difference between the EGRET and baseline edge weights for a particular TF-gene edge. However, because of the manner in which the EGRET prior is created—by penalizing edges involving a TF motif that contains an eQTL variant with a significant negative QBiC effect—only regulatory disruptions are modeled and not the creation of “new” regulatory relationships where none potentially existed before (see Supplemental Note S2).

### EGRET finds regulatory differences between two genetically distinct cell lines

We tested whether EGRET could distinguish patterns of differential gene regulation by comparing two EGRET networks reconstructed from two blood-derived cell lines: GM12878 and K562. We chose these cell lines because high-quality genome sequences are available for both cell lines (Eberle et al. 2017; Zhou et al. 2019), providing high-confidence variant calls; this was especially useful for K562 because the cell line is aneuploid (Zhou et al. 2019). In addition, both cell lines have had relatively large numbers of TFs mapped by ChIP-seq (110 TFs for GM12878 and 204 TFs for K562 in the ReMap 2018 database [Chèneby et al. 2018]), allowing us to use differential TF binding as a way of validating regulatory differences. These cell lines may have many regulatory differences that are due to difference in cell type of origin and not differences in genotype. However, in previous work comparing TF regulatory networks from related tissues (Sonawane et al. 2017), we have observed a higher number of shared edges across tissues than expected by chance. As a result of this and the high-fidelity data available for these cell lines, we felt a comparison of the two cell line networks would provide useful insights despite the limitations listed above.

To build genotype-specific EGRET priors (*E*) for GM12878 and K562, respectively, we generated a reference motif prior *M* using FIMO (Grant et al. 2011) identifying TF motifs in the promoter regions of genes ([−750, +250] relative to transcription start sites) and modified this using eQTL data for lymphoblastoid cell lines (LCLs) from GTEx (Lonsdale et al. 2013), the cell lines’ respective genotypes, and SNP effect predictions from QBiC (Supplemental Notes S1–S3). Comparing edges in *E* against *M* predicted 1520 genotype-altered prior edges for GM12878 and 1182 for K562 (Supplemental Fig. S3) out of a total of 39,690,052 possible edges.

Next, we used human protein–protein interactions between TFs from STRINGdb (Mering et al. 2003) (as used by Sonawane et al. 2017) to construct *P*, and LCL gene expression data from GTEx to construct *C* (Supplemental Note S4). Genes and TFs with low expression, defined as having nonzero values in <50 samples, were filtered out. Performing message passing between *E*, *P*, and *C* produced the final genotype-specific EGRET networks E* for GM12878 and K562 (Supplemental Note S5). For comparison, we constructed a baseline GRN using *M* as input to the message passing with *P* and *C*. We calculated the edge disruption score for each TF-gene pair in each cell line's EGRET network. Because of the relatively small number of genotype-altered edges in the EGRET priors, the majority of edge disruption scores are very close to zero in both cell lines (Supplemental Fig. S4).

For each cell line, we compared both EGRET's predictions of TF binding and the baseline networks to an empirical network based on ChIP-seq data (Supplemental Note S7.1; Chèneby et al. 2018). At multiple cutoffs for the edge disruption scores, EGRET networks outperformed the baseline network prediction of TF binding for variant-disrupted edges (Supplemental Tables S5, S6; Supplemental Note S7.2). Based on these analyses, we considered variant-impacted scores to be those ≥0.35 (Supplemental Table S2).

To capture changes in the disruption score between different genotype-specific networks, we calculated a “regulatory difference score” $R_{ij}^{\left(E\right)}$ (Supplemental Table S4) for each edge between genotypes GM12878 (*g*) and K562 (*k*), defined as

$$R_{ij}^{\left(E\right)}=|d_{g_{ij}}^{\left(E\right)}-d_{k_{ij}}^{\left(E\right)}|.$$

The magnitude of this score is the difference in edge disruption scores between GM12878 $d_{g_{ij}}^{\left(E\right)}$ and K562 $d_{k_{ij}}^{\left(E\right)}$ and reflects the assumption that genetic differences between cell lines will cause differences in predicted regulatory TF-gene interaction strength. A high value of $R_{ij}^{\left(E\right)}$ suggests a difference in the edge disruption scores for the edge *TF*_*i*_ − *G*_*j*_ between the two cell lines, which is interpreted as that relationship being disrupted in one cell line but not the other. Conversely, a value of $R_{ij}^{\left(E\right)}$ close to zero indicates that the edge disruption scores for the edge *TF*_*i*_ − *G*_*j*_ are similar and therefore that the regulatory relationship is either disrupted in both cell lines or remains unaltered in both cell lines.

We again used the cell line–specific ChIP-seq regulatory networks (Supplemental Note S7.1) to construct a *differential ChIP-seq regulatory network* by taking the absolute value of the difference between the GM12878 ChIP-seq network and the K562 ChIP-seq network. This allows us to assign a score of 1 to edges that show differential TF binding (a TF binds the promoter region of a gene in one cell line but not the other) and a score of 0 to edges that show the same pattern of TF binding (a TF either binds the promoter region of a gene in both cell lines, or neither). This scoring allows us to validate the framework modeled by the regulatory difference score $R_{ij}^{\left(E\right)}$. We found that edges with high $R_{ij}^{\left(E\right)}$ scores (those in the top 10%) were enriched for edges showing differential TF binding in the differential ChIP-seq regulatory network (Fisher's exact test *P*-value = 2.4 × 10^−226^); this enrichment was also observed when we considered all scores using a *t*-test (*t*-test *P*-value = 2.296 × 10^−7^).

**Figure 2.**
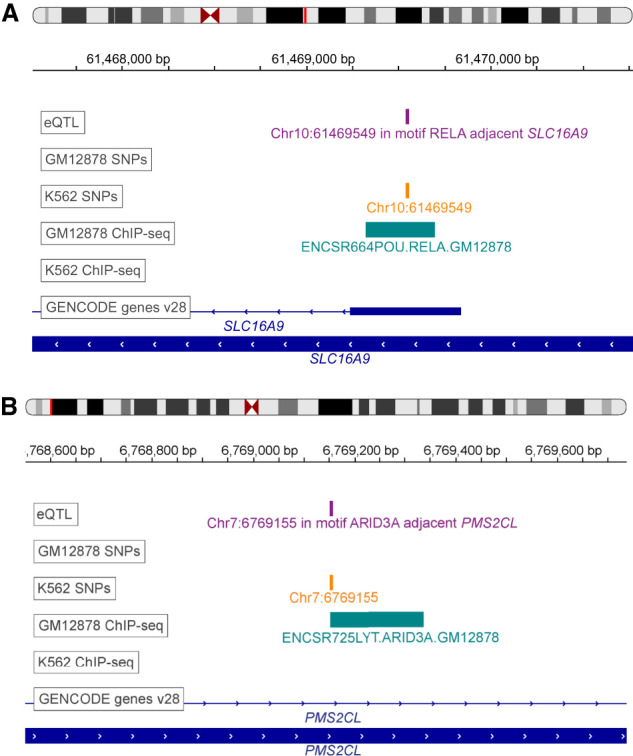
EGRET identifies likely variant-impacted TF binding disruptions. (*A*) Example of RELA binding in K562 but not in GM12878. Positions of eQTLs (purple track), genetic variants (orange track), ChIP-seq binding regions from ReMap2 (teal track) (Chèneby et al. 2018), and genes (blue track) are shown in the region of *SLC16A9*. (*B*) Example of ARID3A binding in K562 but not in GM12878. Positions of eQTLs (purple track), genetic variants (orange track), ChIP-seq binding regions from ReMap2 (teal track) (Chèneby et al. 2018), and genes (blue track) are shown in the region of *PMS2CL*. The eQTL track is labeled according to the TF motif in which the eSNP resides as well as the adjacent eGene.

We highlight two examples of promoter binding of TFs identified through the EGRET network analysis that are likely genotype-specific. First, the edge between the TF RELA and the gene *SLC16A9* (*ENSG00000165449*) has a regulatory difference score of 6.099744, with $d_{g_{ij}}^{\left(E\right)}=0.000256$ in GM12878 and $d_{k_{ij}}^{\left(E\right)}=6.1$ in the K562. These scores suggest that the binding of RELA to the promoter region of *SLC16A9* is disrupted in K562 but not in GM12878. The positions of eQTLs, genetic variants, and ChIP-seq binding regions for RELA in both genotypes (Fig. 2A) indicate that an eQTL variant is present in the promoter region of *SLC16A9* (purple track in Fig. 2A), is associated with the expression of SLC16A9, resides within a RELA binding motif, and is predicted by QBiC to affect the binding of RELA at that location; the disrupting variant is present only in K562 (orange track in Fig. 2A) and not in GM12878; this prediction is supported by the presence of a RELA ChIP-seq binding range in GM12878 but not in K562 (teal track in Fig. 2A). As a second example, consider the edge between of the TF ARID3A and the gene *PMS2CL* (*ENSG00000187953*), with a regulatory difference score of 1.0564 and $d_{g_{ij}}^{\left(E\right)}=0.0096$ in GM12878 and $d_{k_{ij}}^{\left(E\right)}=1.066$ in K562, suggesting that the binding of ARID3A to the promoter region of *PMS2CL* is disrupted in K562 but not in GM12878. This prediction is supported by ChIP-seq-derived TF binding data in the region (Fig. 2B). LocusZoom plots of these regions can be seen in Supplemental Figures S5 and S6. Both of these examples of likely genetic disruptions of TF binding are within the top 20 edge disruption scores for K562 for edges with confirmed differential binding between K562 and GM12878 ChIP-seq experiments.

Because an edge in an EGRET network implies a *regulatory* relationship, as opposed to simply presence of binding by a TF, we wanted to further test our network predictions against assays that captured changes in gene regulation. To do this, we defined a gene-level regulatory difference score $R_{j}^{\left(G\right)}$, which is the sum of regulatory difference scores impinging on the gene $\left(\sum_{i}R_{ij}^{\left(E\right)}\right)$. To test this metric, we used data from an in vitro allele-specific expression (ASE) assay (Biallelic Targeted Self-Transcribing Active Regulatory Region sequencing—BiT-STARR-seq) performed in LCLs (Supplemental Note S7.3; Kalita et al. 2018). We calculated regulatory difference scores per gene $\left(R_{j}^{\left(G\right)}\right)$ and found that the 101 genes having high (top 10%) $R_{j}^{\left(G\right)}$ were enriched for genes harboring ASE-causing variants located within promoter region TF motifs (Fisher's exact test *P*-value = 2.5 × 10^−3^) (see Supplemental Table S2 for details). As a second independent validation, we compared data from a published chromatin accessibility QTL (caQTL) analysis in LCLs (Banovich et al. 2018) to the genes with high regulatory difference scores (again, top 10%) and found that these genes having high $R_{j}^{\left(G\right)}$ values were enriched for having caQTLs within motifs in their promoter regions (Fisher's exact test *P*-value = 1.4 × 10^−4^) (see Supplemental Note S7.4 and Supplemental Table S2 for details). This suggests that many of the predicted regulatory SNPs alter their associated regulatory networks by affecting chromatin accessibility. It is worth noting that our results are based only on the genotypes of two cell lines; we anticipate that using a larger number of genotyped cell lines with available ChIP-seq, caQTL, and ASE data would increase both the specificity and sensitivity of predicting genotype-mediated effects in gene expression.

Overall, these results indicate that EGRET is capable of synthesizing diverse sources of data to model gene regulatory processes and can predict genotype-associated patterns of gene regulation.

### Data integration is required for accurate prediction of regulatory networks

We ran a selection of EGRET versions in order to investigate the contributions of different data types that EGRET uses. First, we ran EGRET using data for GM12878, leaving out the gene expression and PPI information in the message passing, and assessed the ability of the resulting EGRET edge weights to predict ChIP-seq-derived TF binding in GM12878 for the subset of the network where ChIP-seq data were available, using the ChIP-seq regulatory network described in Supplemental Note S7.1. EGRET edge weights using all data types showed almost 5% improvement in ROC-AUC for predicting the validation ChIP-seq network when compared with the EGRET network run without expression and PPI data in the message passing.

We also tested versions of EGRET that left out data types from the prior modification. For example, we ran a version of EGRET, leaving out QBiC effects, thus only requiring genetic variants to be eQTLs and not requiring those variants to have significant QBiC effects. Similarly, we ran a version leaving out eQTL data, only requiring variants in an individual to have a significant QBiC effect, and not requiring the variants to be eQTLs. In validating the ability of disrupted edges $\left(d_{x_{ij}}^{\left(E\right)}\geq 0.35\right)$ to predict the corresponding ChIP-seq edges, we found that all data types were crucial in improving the accuracy of prediction and that leaving out either eQTLs or QBiC effects was detrimental to the validity of the network structure (Supplemental Fig. S7). Further sensitivity analysis of EGRET to different input parameters can be found in Supplemental Note S8 and Supplemental Figures S8 and S9.

### EGRET networks for a population of individuals identify cell type–specific disease associations

A growing body of work indicates that cell type–specific gene regulatory processes affect gene expression (Sonawane et al. 2017; Lopes-Ramos et al. 2018) and do so in a manner dependent on an individual's genotype (Fagny et al. 2017; The GTEx Consortium 2020; Kim-Hellmuth et al. 2020), resulting in changes that alter the structure of functional “communities” or “modules” comprised of TFs and genes, and are enriched for genes associated with tissue-specific biological processes (Padi and Quackenbush 2018). Banovich et al. (2018) had previously analyzed RNA-seq data derived from three cell types: lymphoblastoid cell lines, induced pluripotent stem cells (iPSCs), and cardiomyocytes (CMs; differentiated from the iPSCs). They demonstrated that genes preferentially expressed in CMs were enriched for processes associated with coronary artery disease, and those enriched in LCLs were associated with immune-related conditions. Within this data set, there was a large proportion of cell type–specific eQTLs (Supplemental Fig. S10), and our working hypothesis was that these effects should be linked to cell type–specific regulatory processes affected by an individual's genetic background.

**Figure 3.**
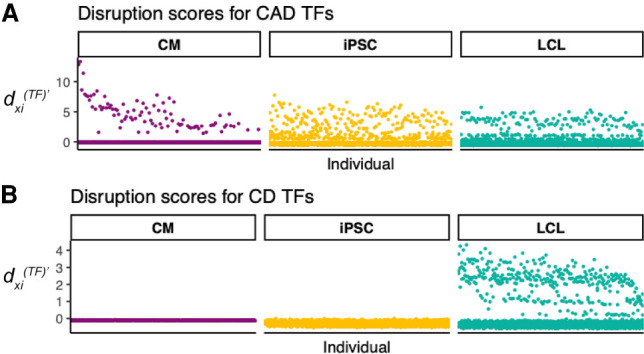
Disease-related TFs are disrupted in relevant cell types. Scaled TF disruption scores $d_{xi}^{(TF)\prime }$ are shown for 119 Yoruba individuals for TFs associated with coronary artery disease (CAD) (*A*) or Crohn's disease (CD) (*B*). Each point represents the scaled TF disruption score for a disease-related (CD or CAD) TF, for a given individual for a given cell type (LCL, CM, or iPSC). Disease-related TFs were identified using the GWAS catalog (Buniello et al. 2019). Scaled TF disruption scores for CAD-related TFs are highest in the cardiac-related cell type (CM). Scaled TF disruption scores for CD-related TFs are highest in the immune cell type, LCL.

To test this, we constructed 357 individual-specific EGRET networks using expression, genotype, and eQTL data from 119 Yoruba individuals for all three cell types used in the Banovich et al. (2018) study (Supplemental Note S9). We also constructed a baseline GRN for each cell type (Supplemental Note S9.1). We calculated TF disruption scores (defined in Supplemental Table S4) for each TF in each individual EGRET network to identify TFs whose regulatory influence was disrupted by variants. TF disruption scores $\left(d_{x_{i}}^{\left(TF\right)}\right)$ were then scaled per individual and cell type to have a mean of zero and standard deviation of one and are denoted $d_{xi}^{(TF)\prime }$ (Supplemental Note S9.2). We then labeled TFs as associated with Crohn's disease (CD) and coronary artery disease (CAD) (Supplemental Tables S7, S8; Supplemental Fig. S11) based on annotation from the NHGRI-EBI GWAS catalog (Buniello et al. 2019). We tested to see whether disease-associated TFs were more likely to have significant disruption scores in relevant cell types. Using a *t*-test, we found that TF disruption scores were significantly higher in cardiomyocytes for TFs associated with CAD than were disruption scores for non-CAD-related TFs (*P* = 4.5256 × 10^−6^); this CAD enrichment was not observed in LCLs (*P* = 0.99831). Similarly, we found TF disruption scores in LCLs, but not CMs, were substantially higher for TFs linked to CD than for non-CD-linked TFs (*P* = 5.3374 × 10^−16^ in LCL networks, *P* = 1 in CM networks) (Table 2). This analysis leads to an important observation: Genotype-mediated, disease-related TF disruptions are cell type–specific and can be identified using networks inferred by EGRET. Indeed, we found that the highest TF disruption scores for CAD TFs occur in CMs (Fig. 3A) and that the highest TF disruption scores for CD TFs occur in LCLs (Fig. 3B).

**Table 2.**
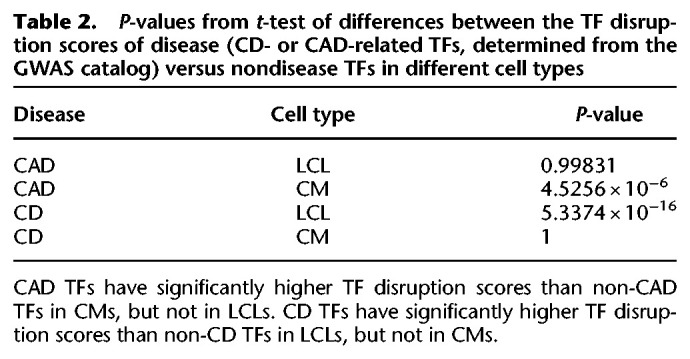
*P*-values from *t*-test of differences between the TF disruption scores of disease (CD- or CAD-related TFs, determined from the GWAS catalog) versus nondisease TFs in different cell types

Further supporting this observation, the TF disruption signal in CAD is dominated in a subset of the study population by a single TF, ERG, which is a member of the erythroblast transformation-specific (ETS) gene family and known to be involved in angiogenesis (Shah et al. 2016). In these individuals, the high TF disruption scores for CAD TFs in CMs are driven by the presence or absence of a mutation on Chromosome 1 (Chr 1: 201,476,815, an eQTL for *CSRP1*) that lies in the binding motif for the TF ERG in the promoter region of the gene *CSRP1* (*ENSG00000159176*). Whereas ERG is identified as CAD-related in the GWAS catalog (Supplemental Fig. S12), *CSRP1* (synonym *CRP1*) is not. However, *CSRP1* is a known smooth muscle marker (Henderson et al. 1999) and has been found by GTEx (Lonsdale et al. 2013) to be highly expressed in smooth muscles, especially in arteries (Supplemental Fig. S13). *CSRP1* has also been associated with the bundling of actin filaments (Tran et al. 2005), cardiovascular development (Chang et al. 2003), and with response to arterial injury (Lilly et al. 2010). Furthermore, knockdown of *CSRP1* in zebrafish caused cardiac bifida (Miyasaka et al. 2007), and a frameshift mutation in *CSRP1* has been linked to congenital cardiac defects in a large human pedigree (Kamar et al. 2017). The results of our EGRET analysis support a previously unreported mechanism of action for ERG that may be disrupted in heart disease—that ERG regulates the expression of *CSRP1* and that this regulation can be disrupted by genetic variation. Specifically, we hypothesize that the SNP located at Chr 1: 201,476,815 is related to CAD because it both influences the binding site of ERG and is an eQTL for a gene involved in related phenotypes (*CSRP1*).

We also tested the hypothesis that the network effects of genetic variants have the potential to subtly change the modular structure of genotype-specific networks, altering the functional network modules active in an individual. ALPACA (Padi and Quackenbush 2018) is a method that compares the modular structure of two networks and identifies modules that differ between the networks. The resulting gene differential modularity (DM) scores indicate which genes have undergone the greatest change in their “modular environment.” We used ALPACA to compare the modular structure of the cell type– and individual-specific EGRET GRNs with the baseline GRN for the corresponding cell type and calculated the DM score for each gene in each network (Supplemental Note S9.3; Supplemental Fig. S14).

**Figure 4.**
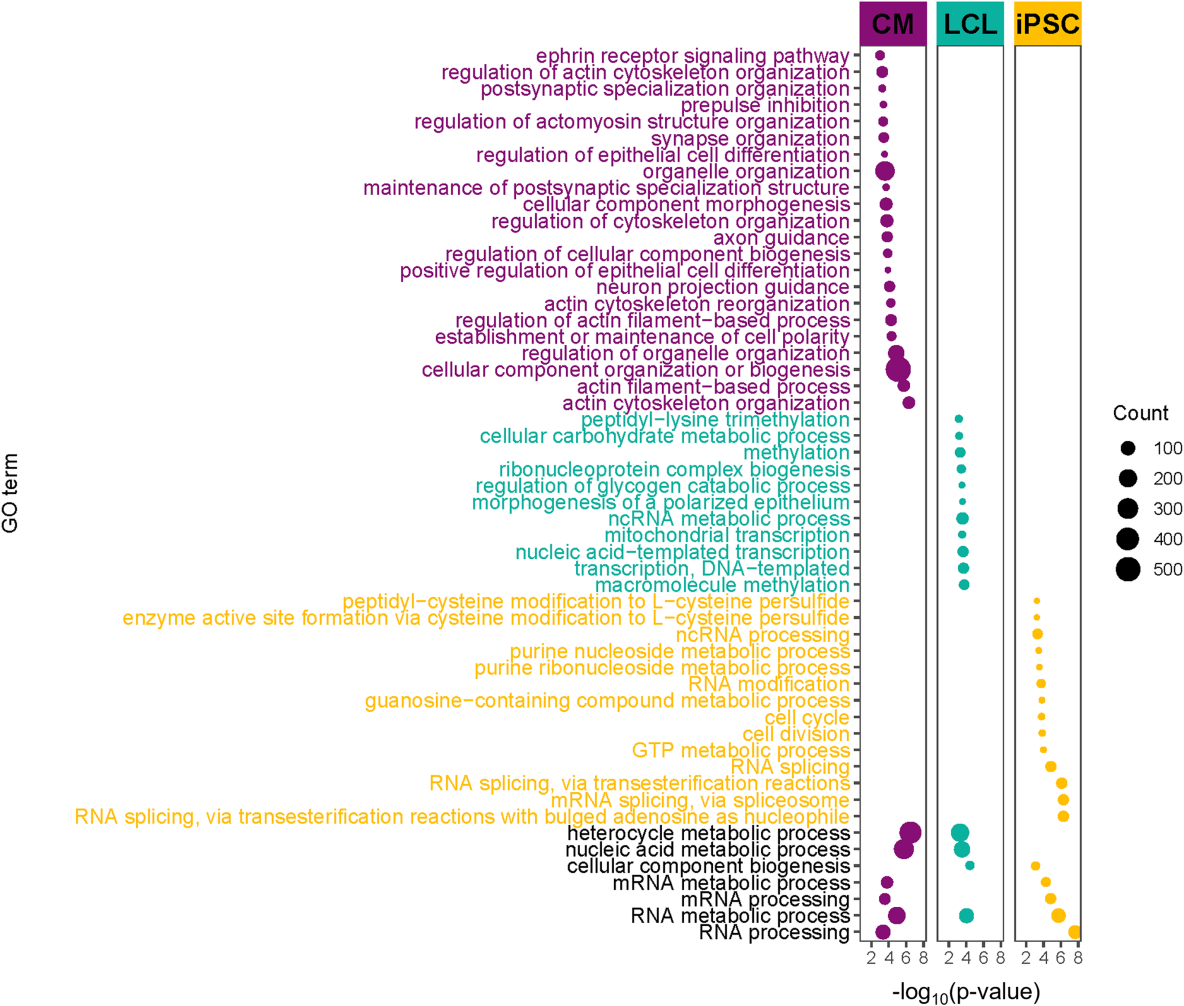
Variant-disrupted gene regulation affecting network modularity is enriched for coronary-/heart-related functions in CMs for an individual with a CAD disruption signature. GO terms enriched in genes with high DM scores for individual 18, the individual with the highest TF disruption score for ERG. Several GO terms related to coronary/cardiac function are enriched in highly ranked DM genes in CMs but not in LCLs and iPSCs. Point size corresponds to the number of high-DM genes annotated with the corresponding GO term. For display purposes, several generic GO terms enriched only in CMs were omitted in this figure. The entire set of enriched GO terms can be seen in Supplemental Figure S18, as well as Supplemental Tables S9–S11.

**Figure 5.**
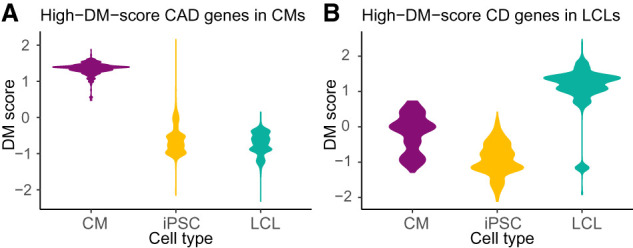
Variant-disrupted disease genes affect the modularity of the individual's regulatory network in the relevant cell type. Differential modularity (DM) scores indicate the extent to which a gene's modular environment in the network changes between the genotype-specific EGRET network and the genotype-agnostic network. (*A*) CAD-related genes with high DM scores in cardiomyocytes (CMs) have low DM scores in the other cell types. (*B*) CD-related genes with high DM scores in LCLs have low scores in the other cell types (see Supplemental Table S2).

Given that individual 18 had the greatest TF disruption score for ERG in CMs, we further investigated cellular processes predicted by EGRET to be variant-perturbed within this individual's three cell type–specific EGRET networks. For each cell type, we ranked this individual's genes by their DM scores from highest to lowest in each cell type, reflecting their predicted impact on altering the modular structure of each cell type–specific network. We used GOrilla (Supplemental Note S9.4; Supplemental Table S2; Eden et al. 2009) with these ranked lists to identify Gene Ontology (GO) biological process functions associated with modules altered by the presence of specific genetic variants. Several GO terms relevant to CMs and cardiovascular functioning and development, including “regulation of actomyosin structure organization,” “prepulse inhibition,” “ephrin receptor signaling pathway,” “maintenance of postsynaptic specialization structure,” and “actin cytoskeleton reorganization” were enriched in CMs from this individual (Fig. 4; Supplemental Table S9) but not in their LCLs or iPSCs (Fig. 4; Supplemental Tables S10, S11). For full enrichment results, see Supplemental Note S9.4 and Supplemental Figures S15–S21. Further evidence of cell type–specific alteration of functional modules can be seen by examining the DM scores of disease-associated target genes (as annotated by the NHGRI-EBI GWAS catalog [Buniello et al. 2019]). Coronary artery disease genes with high DM scores in CMs had low DM scores in iPSCs and LCLs (Fig. 5A). In contrast, genes associated with Crohn's disease, which has a strong immune component, that had high DM scores in LCLs had low DM scores in iPSCs and CMs (Fig. 5B).

EGRET also predicts dosage effects of regulatory SNP variants on network structure. Consider *CSRP1*, which we previously discussed as having a regulatory SNP in its promoter region that can affect binding of the transcription factor ERG. EGRET shows that the presence of a genetic variant in the promoter region of *CSRP1* affects not only regulation by ERG (as seen by a substantial TF disruption score) but also the role that *CSRP1* plays in altering the functional modules in cardiomyocyte GRN models. As seen by *CSRP1*’s DM scores in Supplemental Figure S22, EGRET predicts that the genetic variant exerts its influence on network structure in a dosage-specific manner; individuals homozygous for the disrupting variant are predicted to exhibit the greatest impact on the modularity, those who are heterozygous predicted to have an intermediate effect, and those homozygous for the wild-type predicted to exhibit minimal or no effect on modularity.

Collectively, these results suggest that phenotype- and disease-associated variants can act through disruption of TF binding leading to regulatory changes that manifest themselves both through altered expression of specific target genes and the modification of GRN functional modular structure.

## Discussion

One of the fundamental tenets of genetics is that genotype influences phenotype. For many traits, especially those related to human disease, this connection is not straightforward. The vast majority of phenotype-associated genetic variants are noncoding and have small effect sizes (Hall et al. 2016; Gallagher and Chen-Plotkin 2018), and a recent analysis found that most (71%–100%) 1-Mb windows in the genome contribute to schizophrenia heritability (Loh et al. 2015). This suggests that many variants must act in concert to produce complex trait phenotypes, but the mechanisms by which they exert their influence are unclear. Functional genomics studies have provided some insights into roles of these variants: Variants are enriched in regulatory elements (Maurano et al. 2012; Farh et al. 2015; Vierstra et al. 2020); disease heritability tends to be enriched in tissues relevant to the disease (Boyle et al. 2017); and TF binding plays an important role in explaining heritability of human traits (van de Geijn et al. 2020). Despite this progress at the population level, questions remain regarding the influence of an individual's genotype on these regulatory processes. Answers to these questions will be important for translating population-level insights into clinically actionable information.

EGRET is the first method, to our knowledge, that directly addresses these issues. EGRET begins with a reference motif prior network based on mapping transcription factor binding sites to the regulatory regions of genes. EGRET extends this by modifying the reference motif network based on evidence that SNPs in a gene's regulatory region may influence TF binding as well as gene expression. Subsequently, EGRET uses a previously developed message passing framework (Glass et al. 2013) to iteratively seek consistency between a genotype-altered regulatory network model, TF–TF protein–protein interaction data (acknowledging that TFs can form protein–protein complexes), and gene coexpression data (based on the assumption that genes regulated by the same TFs are likely to exhibit correlated expression). The coexpression matrix is not used to impart any individual specificity to the EGRET networks but to obtain an initial estimate of genes that are likely to be coregulated by TFs. EGRET then outputs edge weights for all TF-to-gene edges. These edge weights reflect the confidence of a regulatory relationship between a TF and gene, given an individual's genotype. We demonstrate EGRET using publicly available eQTL, gene expression, and PPI data and show that the algorithm provides powerful insights regardless of whether or not the individual genotypes are sample matched with other data types. A limitation to the data-integration approach used in EGRET is the difficulty of establishing formal statistical testing frameworks. Because of the variety of input data types, the complex nature of message passing, and genome-wide network scale, estimating uncertainty for these networks remains an open and important challenge.

We validated EGRET in two ways. In the first, we inferred genotype-specific gene regulatory networks for two genotyped cell lines and identified genes that differed in their TF-gene edges, meaning that the model predicts differences in binding of specific TFs to upstream regions of individual genes. When we cross-referenced EGRET's predictions with ChIP-seq data for these cell lines, we found concordance between the predictions and ChIP-seq data, demonstrating that EGRET was able to accurately identify different TF binding patterns and effectively altered the structure of the regulatory network. We also found that genes with high regulatory difference scores between the two cell lines—those predicted to be differently regulated by EGRET—were enriched for QTLs associated with chromatin accessibility and enriched for allele-specific expression, suggesting that the EGRET-predicted regulatory changes are likely to have broader regulatory effects.

Our second validation looked at three different cell types in 119 genotyped individuals. We found distinct cell type–specific and genotype-specific differences in the gene regulatory networks that were linked to disease. Most notable among these were regulatory differences associated with Crohn's disease in lymphoblastoid cell lines and others linked to coronary artery disease in the regulatory networks in cardiomyocytes. Not only were individual TF-gene connections disrupted, but these disruptions led to higher-order changes in the network community structure, reorganizing the network in ways that predict changes in cell type– and disease-specific functional network communities. Our finding of enrichment of CAD-related signals in CM cells supports the results from Banovich et al. (2018), who found that genes more specifically expressed in CMs were enriched in GWAS signals for CAD. However, one should note that it is possible that the enrichment of TFs related to CAD, a vascular disease, could be driven by the limited number of cell types available for study and the fact that the similarity of expression profiles of smooth muscle cells of arteries could be simply more similar to that of CMs than that of LCLs. Taken together, these results from EGRET present a compelling picture of the way in which small-effect, noncoding SNPs work together to influence phenotype. These SNPs have the potential to subtly alter the binding of TFs to their target genes. The direct effect of these individual SNPs is to alter which TFs regulate specific genes. However, their indirect, and possibly more important effect, is to alter the structure and membership of functional communities in the overall regulatory networks. Indeed, it is known that even a small number of TF-gene regulatory edge additions or deletions can lead to significant changes in network modular organization (Padi and Quackenbush 2018).

EGRET is capable of inferring gene regulatory networks specific to an individual's genotype, synthesizing genetic and gene expression data in a way that, for the first time, allows verifiable, disease-associated regulatory changes to be inferred for individual research subjects. As such, EGRET has the potential to substantially advance our understanding of genetic effects on disease risk, development, and response to therapeutic interventions. EGRET currently only makes use of significant eQTLs, which limits the method to common variants. For a future version of EGRET, we would like to include estimates of rare variant effects from methods such as RIVER (Li et al. 2017). Potential applications of EGRET are wide ranging. EGRET can be used to infer a specific gene regulatory network for any individual for whom genotype data are available, even without associated gene expression data—provided there are expression and eQTL data from a relevant cell type obtained from a sufficiently large population to infer accurate regulatory network models. This implies that EGRET can be used in interpreting disease-linked variation where the variant colocalizes with an eQTL signal, provided that the eQTL signal overlaps with a TF motif and there is an effect on that motif as estimated by QBiC.

Given EGRET's ability to synthesize both *cis-* and *trans-*regulatory information, EGRET edge weights may also be useful for improving gene expression prediction models. Recent work by Patel and Bush (2021) has shown that TF regulatory networks reconstructed using the underlying message passing approach (Glass et al. 2013) for EGRET improve prediction of gene expression compared with other baseline models. EGRET can also be used to retrospectively analyze large cohort GWAS studies to tease out mechanistic associations for phenotype-linked genetic variants, as well as in the context of new studies that seek to understand disease mechanisms and the regulatory role of noncoding genetic variants.

## Methods

To generate the reference motif prior network, we scanned the hg19 human reference assembly for the presence of TF motifs using FIMO (Grant et al. 2011) and applying a *P*-value cutoff of 10^−4^. Motifs that were present within the promoter regions of genes were selected to construct *M* (Supplemental Note S1). We chose to use the hg19 reference genome because, at the time of analysis, all of the eQTL data used, including the latest version of GTEx (v7 at the time), as well as the Banovich et al. (2018) data were mapped to hg19. Since completion of this analysis, version 8 of GTEx has been released, which is mapped to GRCh38 (hg38), and we would recommend using the latest version of GTEx and the most current genome build (hg38) for future analyses. By design, EGRET can be applied to data of any reference genome provided that the positions of genome variants, eQTLs, gene promoters, and motifs are all mapped to the same reference genome. However, we note that, whereas there are some differences between hg19 and hg38, the key elements for EGRET, including mapping of SNPs to promoter regions, are largely unchanged, with 99% of SNPs from hg19 mapping to hg38 and most discordant SNPs being low-confidence (Pan et al. 2019). Expression QTLs for LCLs from GTEx version 7 (Lonsdale et al. 2013) were downloaded from https://gtexportal.org/home/datasets and filtered to select eQTLs where the variant resided within a TF motif within a promoter region *and* where the eGene was the gene adjacent to (and associated with) the promoter. Genotypes for NA12878 (corresponding to the GM12878 cell line) and K562 were downloaded from https://www.illumina.com/platinumgenomes.html and https://www.encodeproject.org/files/ENCFF538YDL/, respectively. Using the eQTL variants within motifs, we selected those variants where at least one of the cell lines (K562 or GM12878) had at least one alternate allele of the eQTL variant. QBiC (Martin et al. 2019) was then run on these eQTLs, using hg19 as a reference genome (Supplemental Note S2). A modified EGRET prior *E* was then defined as

$$E_{ij}=M_{ij}-\underset{s}{\sum }|q_{s_{ij}}A_{s_{ij}}{\text{β}}_{s_{ij}}|,$$

where $A_{s_{ij}}$ is the alternate allele count of the individual at that location, ${\text{β}}_{s_{ij}}$ is the beta value of the eQTL, and $q_{s_{ij}}$ is the QBiC effect of the SNP on the binding of the TF corresponding to the motif in which the variant resides (Supplemental Note S3). Gene expression data as TPMs (transcripts per million) for lymphoblastoid cell lines from the Genotype-Tissue Expression Project (GTEx) version 7 (Lonsdale et al. 2013) were downloaded from https://gtexportal.org/home/datasets and pruned to keep only genes that had nonzero expression values in at least 50 samples. The protein–protein interaction network used in Sonawane et al. (2017) was then filtered to keep only proteins whose corresponding genes met the same expression requirements described above (nonzero expression values in at least 50 samples) (Supplemental Note S4). The message passing framework (Glass et al. 2013) from the pandaR package was then used to combine the EGRET prior *E*, PPI prior *P*, and gene expression data *C*, resulting in a predicted genotype-specific gene regulatory network for an individual (Supplemental Note S5).

Additional detail regarding the methods is available in the Supplemental Material.

### Software availability

The analysis presented here uses publicly available data sources as outlined in the Methods. The network models inferred using EGRET and presented here have been deposited into the GRAND database (Ben Guebila et al. 2022; https://grand.networkmedicine.org/downloads/) and are freely available for download (search “EGRET”). An implementation of EGRET in R (R Core Team 2019) is available through the Network Zoo R package (netZooR v0.9; https://netzoo.github.io/zooanimals/egret/) with a step-by-step tutorial. EGRET analysis scripts from this work are provided as Supplemental Code.

## Supplementary Material

Supplemental Material

## References

[GR275107WEIC1] Banovich NE, Li YI, Raj A, Ward MC, Greenside P, Calderon D, Tung PY, Burnett JE, Myrthil M, Thomas SM, 2018. Impact of regulatory variation across human ipscs and differentiated cells. Genome Res 28: 122–131. 10.1101/gr.224436.117 29208628 PMC5749177

[GR275107WEIC2] Barbeira AN, Bonazzola R, Gamazon ER, Liang Y, Park Y, Kim-Hellmuth S, Wang G, Jiang Z, Zhou D, Hormozdiari F, 2021. Exploiting the GTEx resources to decipher the mechanisms at GWAS loci. Genome Biol 22: 49. 10.1186/s13059-020-02252-4 33499903 PMC7836161

[GR275107WEIC3] Basso K, Margolin AA, Stolovitzky G, Klein U, Dalla-Favera R, Califano A. 2005. Reverse engineering of regulatory networks in human B cells. Nat Genet 37: 382–390. 10.1038/ng1532 15778709

[GR275107WEIC04] Ben Guebila M, Lopes-Ramos CM, Weighill D, Sonawane AR, Burkholz R, Shamsaei B, Platig J, Glass K, Kuijjer ML, Quackenbush J. 2022. GRAND: a database of gene regulatory network models across human conditions. Nucleic Acids Res 50: D610–D621. 10.1093/nar/gkab778 34508353 PMC8728257

[GR275107WEIC4] Boyle EA, Li YI, Pritchard JK. 2017. An expanded view of complex traits: from polygenic to omnigenic. Cell 169: 1177–1186. 10.1016/j.cell.2017.05.038 28622505 PMC5536862

[GR275107WEIC5] Buniello A, MacArthur JAL, Cerezo M, Harris LW, Hayhurst J, Malangone C, McMahon A, Morales J, Mountjoy E, Sollis E, 2019. The NHGRI-EBI GWAS catalog of published genome-wide association studies, targeted arrays and summary statistics 2019. Nucleic Acids Res 47: D1005–D1012. 10.1093/nar/gky1120 30445434 PMC6323933

[GR275107WEIC6] Chang DF, Belaguli NS, Iyer D, Roberts WB, Wu SP, Dong XR, Marx JG, Moore MS, Beckerle MC, Majesky MW, 2003. Cysteine-rich LIM-only proteins CRP1 and CRP2 are potent smooth muscle differentiation cofactors. Dev Cell 4: 107–118. 10.1016/S1534-5807(02)00396-9 12530967

[GR275107WEIC7] Chèneby J, Gheorghe M, Artufel M, Mathelier A, Ballester B. 2018. ReMap 2018: an updated atlas of regulatory regions from an integrative analysis of DNA-binding ChIP-seq experiments. Nucleic Acids Res 46: D267–D275. 10.1093/nar/gkx1092 29126285 PMC5753247

[GR275107WEIC8] Eberle MA, Fritzilas E, Krusche P, Källberg M, Moore BL, Bekritsky MA, Iqbal Z, Chuang HY, Humphray SJ, Halpern AL, 2017. A reference data set of 5.4 million phased human variants validated by genetic inheritance from sequencing a three-generation 17-member pedigree. Genome Res 27: 157–164. 10.1101/gr.210500.116 27903644 PMC5204340

[GR275107WEIC9] Eden E, Navon R, Steinfeld I, Lipson D, Yakhini Z. 2009. *GOrilla*: a tool for discovery and visualization of enriched go terms in ranked gene lists. BMC Bioinformatics 10: 48. 10.1186/1471-2105-10-48 19192299 PMC2644678

[GR275107WEIC10] Fagny M, Paulson JN, Kuijjer ML, Sonawane AR, Chen CY, Lopes-Ramos CM, Glass K, Quackenbush J, Platig J. 2017. Exploring regulation in tissues with eqtl networks. Proc Natl Acad Sci 114: E7841–E7850. 10.1073/pnas.1707375114 28851834 PMC5604022

[GR275107WEIC11] Faith JJ, Hayete B, Thaden JT, Mogno I, Wierzbowski J, Cottarel G, Kasif S, Collins JJ, Gardner TS. 2007. Large-scale mapping and validation of *Escherichia coli* transcriptional regulation from a compendium of expression profiles. PLoS Biol 5: e8. 10.1371/journal.pbio.0050008 17214507 PMC1764438

[GR275107WEIC12] Farh KKH, Marson A, Zhu J, Kleinewietfeld M, Housley WJ, Beik S, Shoresh N, Whitton H, Ryan RJH, Shishkin AA, 2015. Genetic and epigenetic finemapping of causal autoimmune disease variants. Nature 518: 337–343. 10.1038/nature13835 25363779 PMC4336207

[GR275107WEIC13] Finucane HK, Bulik-Sullivan B, Gusev A, Trynka G, Reshef Y, Loh PR, Anttila V, Xu H, Zang C, Farh K, 2015. Partitioning heritability by functional annotation using genome-wide association summary statistics. Nat Genet 47: 1228–1235. 10.1038/ng.3404 26414678 PMC4626285

[GR275107WEIC14] Gallagher MD, Chen-Plotkin AS. 2018. The post-GWAS era: from association to function. Am J Hum Genet 102: 717–730. 10.1016/j.ajhg.2018.04.002 29727686 PMC5986732

[GR275107WEIC15] Glass K, Huttenhower C, Quackenbush J, Yuan GC. 2013. Passing messages between biological networks to refine predicted interactions. PLoS One 8: e64832. 10.1371/journal.pone.0064832 23741402 PMC3669401

[GR275107WEIC16] Grant CE, Bailey TL, Noble WS. 2011. FIMO: scanning for occurrences of a given motif. Bioinformatics 27: 1017–1018. 10.1093/bioinformatics/btr064 21330290 PMC3065696

[GR275107WEIC17] The GTEx Consortium. 2020. The GTEx Consortium atlas of genetic regulatory effects across human tissues. Science 369: 1318–1330. 10.1126/science.aaz1776 32913098 PMC7737656

[GR275107WEIC18] Gusev A, Mancuso N, Won H, Kousi M, Finucane HK, Reshef Y, Song L, Safi A, McCarroll S, Neale BM, 2018. Transcriptome-wide association study of schizophrenia and chromatin activity yields mechanistic disease insights. Nat Genet 50: 538–548. 10.1038/s41588-018-0092-1 29632383 PMC5942893

[GR275107WEIC19] Hall MA, Moore JH, Ritchie MD. 2016. Embracing complex associations in common traits: critical considerations for precision medicine. Trends Genet 32: 470–484. 10.1016/j.tig.2016.06.001 27392675

[GR275107WEIC20] Henderson JR, Macalma T, Brown D, Richardson JA, Olson EN, Beckerle MC. 1999. The LIM protein, CRP1, is a smooth muscle marker. Dev Dyn 214: 229–238. 10.1002/(SICI)1097-0177(199903)214:3<229::AID-AJA6>3.0.CO;2-S 10090149

[GR275107WEIC21] Kalita CA, Brown CD, Freiman A, Isherwood J, Wen X, Pique-Regi R, Luca F. 2018. High-throughput characterization of genetic effects onDNA–protein binding and gene transcription. Genome Res 28: 1701–1708. 10.1101/gr.237354.118 30254052 PMC6211638

[GR275107WEIC22] Kamar A, Fahed AC, Shibbani K, El-Hachem N, Bou-Slaiman S, Arabi M, Kurban M, Seidman JG, Seidman CE, Haidar R, 2017. A novel role for *CRSP1* in a Lebanese family with congenital cardiac defects. Front Genet 8: 217. 10.3389/fgene.2017.00217 29326753 PMC5741687

[GR275107WEIC23] Kim-Hellmuth S, Aguet F, Oliva M, Muñoz-Aguirre M, Kasela S, Wucher V, Castel SE, Hamel AR, Viñuela A, Roberts AL, 2020. Cell type–specific genetic regulation of gene expression across human tissues. Science 369: eaaz8528. 10.1126/science.aaz8528 32913075 PMC8051643

[GR275107WEIC24] Lambert SA, Jolma A, Campitelli LF, Das PK, Yin Y, Albu M, Chen X, Taipale J, Hughes TR, Weirauch MT. 2018. The human transcription factors. Cell 172: 650–665. 10.1016/j.cell.2018.01.029 29425488 PMC12908702

[GR275107WEIC25] Li X, Kim Y, Tsang EK, Davis JR, Damani FN, Chiang C, Hess GT, Zappala Z, Strober BJ, Scott AJ, 2017. The impact of rare variation on gene expression across tissues. Nature 550: 239–243. 10.1038/nature24267 29022581 PMC5877409

[GR275107WEIC26] Lilly B, Clark KA, Yoshigi M, Pronovost S, Wu ML, Periasamy M, Chi M, Paul RJ, Yet SF, Beckerle MC. 2010. Loss of the serum response factor cofactor, cysteine-rich protein 1, attenuates neointima formation in the mouse. Arterioscler Thromb Vasc Biol 30: 694–701. 10.1161/ATVBAHA.109.200741 20056913 PMC2998921

[GR275107WEIC27] Loh PR, Bhatia G, Gusev A, Finucane HK, Bulik-Sullivan BK, Pollack SJ, de Candia TR, Lee SH, Wray NR, Kendler KS, 2015. Contrasting genetic architectures of schizophrenia and other complex diseases using fast variance-components analysis. Nat Genet 47: 1385–1392. 10.1038/ng.3431 26523775 PMC4666835

[GR275107WEIC28] Lonsdale J, Thomas J, Salvatore M, Phillips R, Lo E, Shad S, Hasz R, Walters G, Garcia F, Young N, 2013. The Genotype-Tissue Expression (GTEx) project. Nat Genet 45: 580. 10.1038/ng.2653 23715323 PMC4010069

[GR275107WEIC29] Lopes-Ramos CM, Kuijjer ML, Ogino S, Fuchs CS, DeMeo DL, Glass K, Quackenbush J. 2018. Gene regulatory network analysis identifies sex-linked differences in colon cancer drug metabolism. Cancer Res 78: 5538–5547. 10.1158/0008-5472.CAN-18-0454 30275053 PMC6169995

[GR275107WEIC30] Margolin AA, Nemenman I, Basso K, Wiggins C, Stolovitzky G, Dalla Favera R, Califano A. 2006. ARACNE: an algorithm for the reconstruction of gene regulatory networks in a mammalian cellular context. BMC Bioinformatics 7: S7. 10.1186/1471-2105-7-S1-S7 PMC1810318 16723010

[GR275107WEIC31] Martin V, Zhao J, Afek A, Mielko Z, Gordân R. 2019. QBiC-Pred: quantitative predictions of transcription factor binding changes due to sequence variants. Nucleic Acids Res 47: W127–W135. 10.1093/nar/gkz363 31114870 PMC6602471

[GR275107WEIC32] Maurano MT, Humbert R, Rynes E, Thurman RE, Haugen E, Wang H, Reynolds AP, Sandstrom R, Qu H, Brody J, 2012. Systematic localization of common disease-associated variation in regulatoryDNA. Science 337: 1190–1195. 10.1126/science.1222794 22955828 PMC3771521

[GR275107WEIC33] Mering CV, Huynen M, Jaeggi D, Schmidt S, Bork P, Snel B. 2003. STRING: a database of predicted functional associations between proteins. Nucleic Acids Res 31: 258–261. 10.1093/nar/gkg034 12519996 PMC165481

[GR275107WEIC34] Miyasaka KY, Kida YS, Sato T, Minami M, Ogura T. 2007. Csrp1 regulates dynamic cell movements of the mesendoderm and cardiac mesoderm through interactions with Dishevelled and Diversin. Proc Natl Acad Sci 104: 11274–11279. 10.1073/pnas.0702000104 17592114 PMC2040889

[GR275107WEIC35] Neph S, Stergachis A, Reynolds A, Sandstrom R, Borenstein E, Stamatoyannopoulos J. 2012. Circuitry and dynamics of human transcription factor regulatory networks. Cell 150: 1274–1286. 10.1016/j.cell.2012.04.040 22959076 PMC3679407

[GR275107WEIC36] Padi M, Quackenbush J. 2018. Detecting phenotype-driven transitions in regulatory network structure. NPJ Syst Biol Appl 4: 16. 10.1038/s41540-018-0052-5 29707235 PMC5908977

[GR275107WEIC37] Pan B, Kusko R, Xiao W, Zheng Y, Liu Z, Xiao C, Sakkiah S, Guo W, Gong P, Zhang C, 2019. Similarities and differences between variants called with human reference genome HG19 or HG38. BMC Bioinformatics 20: 17–29. 10.1186/s12859-019-2620-0 30871461 PMC6419332

[GR275107WEIC38] Patel N, Bush WS. 2021. Modeling transcriptional regulation using gene regulatory networks based on multi-omics data sources. BMC Bioinformatics 22: 200. 10.1186/s12859-021-04126-3 33874910 PMC8056605

[GR275107WEIC039] R Core Team. 2019. R: a language and environment for statistical computing. R Foundation for Statistical Computing, Vienna. https://www.R-project.org/.

[GR275107WEIC39] Schaid DJ, Chen W, Larson NB. 2018. From genome-wide associations to candidate causal variants by statistical fine-mapping. Nat Rev Genet 19: 491–504. 10.1038/s41576-018-0016-z 29844615 PMC6050137

[GR275107WEIC40] Shah AV, Birdsey GM, Randi AM. 2016. Regulation of endothelial homeostasis, vascular development and angiogenesis by the transcription factorERG. Vascul Pharmacol 86: 3–13. 10.1016/j.vph.2016.05.003 27208692 PMC5404112

[GR275107WEIC41] Sonawane AR, Platig J, Fagny M, Chen CY, Paulson JN, Lopes-Ramos CM, DeMeo DL, Quackenbush J, Glass K, Kuijjer ML. 2017. Understanding tissue-specific gene regulation. Cell Rep 21: 1077–1088. 10.1016/j.celrep.2017.10.001 29069589 PMC5828531

[GR275107WEIC42] Tran TC, Singleton C, Fraley TS, Greenwood JA. 2005. Cysteine-rich protein 1 (CRP1) regulates actin filament bundling. BMC Cell Biol 6: 45. 10.1186/1471-2121-6-45 16336664 PMC1318456

[GR275107WEIC43] van de Geijn B, Finucane H, Gazal S, Hormozdiari F, Amariuta T, Liu X, Gusev A, Loh PR, Reshef Y, Kichaev G, 2020. Annotations capturing cell type–specific TF binding explain a large fraction of disease heritability. Hum Mol Genet 29: 1057–1067. 10.1093/hmg/ddz226 31595288 PMC7206853

[GR275107WEIC44] Vierstra J, Lazar J, Sandstrom R, Halow J, Lee K, Bates D, Diegel M, Dunn D, Neri F, Haugen E, 2020. Global reference mapping of human transcription factor footprints. Nature 583: 729–736. 10.1038/s41586-020-2528-x 32728250 PMC7410829

[GR275107WEIC45] Westra HJ, Peters MJ, Esko T, Yaghootkar H, Schurmann C, Kettunen J, Christiansen MW, Fairfax BP, Schramm K, Powell JE, 2013. Systematic identification of *trans* eQTLs as putative drivers of known disease associations. Nat Genet 45: 1238–1243. 10.1038/ng.2756 24013639 PMC3991562

[GR275107WEIC46] Zhou B, Ho SS, Greer SU, Zhu X, Bell JM, Arthur JG, Spies N, Zhang X, Byeon S, Pattni R, 2019. Comprehensive, integrated, and phased whole-genome analysis of the primary ENCODE cell line K562. Genome Res 29: 472–484. 10.1101/gr.234948.118 30737237 PMC6396411

[GR275107WEIC47] Zhu Z, Zhang F, Hu H, Bakshi A, Robinson MR, Powell JE, Montgomery GW, Goddard ME, Wray NR, Visscher PM, 2016. Integration of summary data from GWAS and eQTL studies predicts complex trait gene targets. Nat Genet 48: 481–487. 10.1038/ng.3538 27019110

