# Supplementary material for: Predicting genotype-specific gene regulatory networks
Source: Genome Res. 2022 Mar;32(3):524–33. doi: 10.1101/gr.275107.120 (PMC8896459; doi:10.1101/gr.275107.120)
Supplement: Supplemental Material [file supp_32_3_524__DC1.html]

Predicting genotype-specific gene regulatory networks — Supplemental Material 

# Predicting genotype-specific gene regulatory networks

## Supplemental Material

- Supplementary\_Table\_S9.txt
- Supplementary\_Table\_S10.txt
- Supplementary\_Table\_S11.txt
- Supplementary\_Code.zip
- Supplementary\_Material.pdf
