# Supplementary material for: Predicting genotype-specific gene regulatory networks
Source: Genome Res. 2022 Mar;32(3):524–33. doi: 10.1101/gr.275107.120 (PMC8896459; doi:10.1101/gr.275107.120)
Supplement: Supplemental Material [file supp_gr.275107.120_Supplementary_Material.pdf]

Supplementary Material:

Predicting genotype-specific gene regulatory networks

Deborah Weighill, Marouen Ben Guebila, Kimberly Glass, John Quackenbush, John Platig

Contents

Note S1 Reference motif prior 7

Note S2 eQTLs, genotypes and QBiC 7

Note S3 Prior modification 8

Note S4 Gene expression and PPI data 9

Note S5 Message Passing Parameters 9

Note S6 Computational Requirements 10

Note S7 Comparison of EGRET networks from two cell line genotypes 11

- Note S7.1 ChIP-seq regulatory network 11
- Note S7.2 Improving prediction of TF binding 12
- Note S7.3 Allele-specific expression 12
- Note S7.4 Chromatin accessibility QTLs 13

Note S8 Sensitivity analysis 13

Note S9 Population study of 119 individuals across 3 cell types 13

- Note S9.1 Network Construction 13
- Note S9.2 TF disruption scores 14
- Note S9.3 Differential modularity with ALPACA 14
- Note S9.4 Functional Enrichment 15
  - Note S9.4.1 Functional enrichment in genes with high DM scores in individual 18. . . 15
  - Note S9.4.2 Functional enrichment in different communities in individual 18 15

List of Figures

S1 **Diagram illustrating the process and datatypes required for EGRET network construction.** EGRET begins with a reference motif prior representing the presence/absence of TF motifs in the promoter regions of genes. This is then modified by the individual's genetic mutations, penalizing motif-gene edges in which there exists a variant within the TF motif for which the individual has the alternate allele (A), the variant is an eQTL for the adjacent gene (β) and the variant is predicted through QBiC to disrupt TF binding at that location (q). These prior edges are then penalized by the absolute value of the product of the alternate allele count, the QBiC effect, and the eQTL beta value. Message passing then integrates the co-expression network (C) and PPI network (P) with the EGRET prior (E), resulting in a final genotype-specific GRN per individual (E^*). 16

S2 **eQTLs per gene/edge.** (A) Distribution of number of eQTLs which fall within the promoter regions of genes (i.e., fall within any TF motif within the promoter region of a given gene); (B) Distribution of number of eQTLs which fall within a particular TF's motif within a particular gene's promoter; (C) Distribution of number of disruptive (significant negative QBiC effect - see Note S2) eQTLs which fall within the promoter regions of genes (i.e. fall within at least one TF motif within the promoter region of a given gene); (D) Distribution of number of disruptive (significant negative QBiC effect - see Note S2) eQTLs which fall within a particular TF's motif within a particular gene's promoter. . . . 17

S3 **Distribution of non-zero prior modifications** $\sum_s |q_{s_{ij}} A_{s_{ij}} \beta_{s_{ij}}|$ for **(A)** GM12878 and **(B)** K562. 18

S4 **Distribution of edge disruption scores** $d_{x_{ij}}^{(E)}$ **for GM12878 and K562.** **(A)** Violin plot of edge disruption scores. **(B)** Boxplot of $\log_{10}$ disruption scores. 18

S5 ***SLC16A9* region eQTLs.** LocusZoom plot (Boughton et al., 2021) of GTEx LCL eQTLs in the region of *SLC16A9*. 19

S6 ***PMS2CL* region eQTLs.** LocusZoom plot (Boughton et al., 2021) of GTEx LCL eQTLs in the region of *PMS2CL*. 19

S7 **Contribution of different data types to EGRET.** Percentage improvement in the prediction of the ChIP-seq regulatory network by the EGRET network E^* in GM12878, compared to that of the baseline network B^*. Each bar represents the AUC-ROC improvement when using a different combination of data types in the prior modification, for each SNP s with QBiC effect q, alternate allele count A and eQTL beta value β. Percentage improvement calculated as $(AUC_{E^*} - AUC_{B^*})/AUC_{B^*}$ 20

S8 **Sensitivity analysis - motif calls.** Accuracy of the global network structure, validated against the gold-standard ChIP-seq network for GM12878, with different significance thresholds for the motif prior. 20

S9 **Sensitivity analysis - eQTLs.**(A) Accuracy of the global EGRET network, validated against the gold-standard ChIP-seq network for GM12878, with different significance thresholds for calling eQTLs. (B) Accuracy of the variant disrupted edges, validated against the gold-standard ChIP-seq network for GM12878, with different significance thresholds for calling eQTLs. (C) Number of disrupted edges overlapping with ChIP-seq data at different eQTL thresholds. (*) See Note S2 for details on the default GTEx approach for determining the p-value cutoff. 21

S10 **Cell type eQTL overlap.** Venn diagram indicating the overlap of eQTLs between LCL, iPSC and CM cell types in the Banovich et al. dataset. 22

S11 **CAD/CD TF overlap.** Venn diagram indicating the overlap between TFs associated with CAD and CD through GWAS. 23

S12 **GWAS SNP associated with CAD.** Position of SNP rs2836633 which is associated with CAD via a GWAS association. 23

S13 ***CSRP1* expression.** TPM expression level of *CSRP1* (*ENSG00000159176*) across all tissues available in GTEx. Plot obtained from the GTEx portal (Lonsdale et al., 2013). 24

S14 **Differential modularity scores.** Distributions of scaled differential modularity (DM) scores of genes in EGRET networks from 119 Yoruba individuals in three cell types. . . . 24

S15 **Hierarchy of GO terms enriched in the genes with highest DM scores in individual 18 (genotype NA18523) in CMs, based on the mHG score test (Eden et al., 2009). Enrichment performed using GORILLA (Eden et al., 2009).** GO terms are colored according to the significance of the p-value. 25

S16 **Hierarchy of GO terms enriched in the genes with highest DM scores in individual 18 (genotype NA18523) in iPSCs, based on the mHG score test (Eden et al., 2009).** Enrichment performed using GORILLA (Eden et al., 2009). GO terms are colored according to the significance of the p-value. 26

S17 **Hierarchy of GO terms enriched in the genes with highest DM scores in individual 18 (genotype NA18523) in LCLs, based on the mHG score test (Eden et al., 2009).** Enrichment performed using GORILLA (Eden et al., 2009). GO terms are colored according to the significance of the p-value. 27

S18 **GO terms enriched in genes with high DM scores in individual 18**, the individual with the highest TF disruption score for ERG. Point size corresponds to the the number of high-DM genes annotated with the corresponding GO term. 28

S19 **Functional enrichment in community 1.** Functional enrichment in community 1 of individual 18's EGRET network. Enrichment and visualization performed using the g:Profiler R package (Raudvere et al., 2019). 29

S20 **Functional enrichment in community 2.** Functional enrichment in community 2 of individual 18's EGRET network. Enrichment and visualization performed using the g:Profiler R package (Raudvere et al., 2019). 30

S21 **Functional enrichment in community 3.** Functional enrichment in community 3 of individual 18's EGRET network. Enrichment and visualization performed using the g:Profiler R package (Raudvere et al., 2019). 31

S22 **DM scores of *CSRP1* for 119 individuals in Yoruba population.** Point color intensity corresponds to the individual's alternate allele dosage A for the SNP within the ERG binding motif, and point size corresponds to the TF disruption $d_{x_i}^{(TF)'}$ score of ERG. 32

List of Tables

S1 Inputs to EGRET. 33

S2 **Thresholds:** Different thresholds/thresholding strategies used during EGRET network construction and analysis. 34

S3 **Computational requirements:** Statistics on computational requirements for running EGRET. 34

S4 Calculated outputs from EGRET. 35

S5 Improvement in AUC-ROC for the prediction of the ChIP-seq regulatory network in GM12878 when using EGRET edge weights, over using baseline network edge-weights, for different cutoffs of $d_{x_{ij}}^{(E)}$. Total number of negatives (N), total number of positives (P), improvement in the AUC-ROC as well as the Delong p-value for the improvement are reported. 36

S6 Improvement in AUC-ROC for the prediction of the ChIP-seq regulatory network in K562 when using EGRET edge weights, over using baseline network edge-weights, for different cutoffs of $d_{x_{ij}}^{(E)}$. Total number of negatives (N), total number of positives (P), improvement in the AUC-ROC as well as the Delong p-value for the improvement are reported. 37

S7 GWAS catalog study references for CAD genes. 38

S8 GWAS catalog study references for CD genes. 39

S9 See supplementary file attachment Supplementary_Table_S9.txt 40

S10 See supplementary file attachment Supplementary_Table_S10.txt 40

S11 See supplementary file attachment Supplementary_Table_S11.txt 40

Note S1 Reference motif prior

The hg19 human reference assembly was scanned for the presence of TF motifs using FIMO (Grant et al., 2011) and applying a p-value cutoff of 10^{-4}. Motifs that were present within the promoter regions of genes were selected by identifying motifs that overlapped with the 1kb region (-750, +250) around all possible transcription start sites (TSS) of a gene (so that we consider the TSS of each transcript of a gene), making use of the GenomicRanges R package (Lawrence et al., 2013). Transcription start sites for each transcript were downloaded from the UCSC Table Browser `https://genome.ucsc.edu/cgi-bin/hgTables`, in the Ensembl genes table for hg19 on 06/10/2020. The resulting mapped motifs were then collapsed to construct the reference motif prior network M defined as:

$$M_{ij} = \begin{cases} 1 & \text{if motif of TF } i \text{ overlaps with promoter region of gene } j \\ 0 & \text{otherwise} \end{cases}$$

We chose to use the hg19 reference genome because at the time of analysis, all of the eQTL data used, including the latest version of GTEx (v7 at the time), as well as the Banovich et al. (2018) data was mapped to hg19.

Note S2 eQTLs, genotypes and QBiC

Expression QTLs for LCLs from GTEx version 7 (Lonsdale et al., 2013; GTEx Consortium, 2017) were downloaded from `https://gtexportal.org/home/datasets` on 06/10/2020. Determination of eQTLs is described in the original paper from the GTEx consortium (GTEx Consortium, 2017). Briefly, linear regression in the FastQTL (Ongen et al., 2016) package was used to identify cis-eQTLs, while adjusting for several potentially confounding factors, including sex and genotyping platform, among others. Variants within 1 Mb of the TSS of genes were considered. To determine significant variant-gene pairs, the following approach was taken: [Quotation from `https://www.gtexportal.org/home/documentationPage`]. *"a genome-wide empirical p-value threshold, pt, was defined as the empirical p-value of the gene closest to the 0.05 FDR threshold. pt was then used to calculate a nominal p-value threshold for each gene based on the beta distribution model (from FastQTL) of the minimum p-value distribution f(pmin) obtained from the permutations for the gene. Specifically, the nominal threshold was calculated as $F^{-1}(pt)$, where F^{-1} is the inverse cumulative distribution. For each gene, variants with a nominal p-value below the gene-level threshold were considered significant and included in the final list of variant-gene pairs."*

These eQTLs were then filtered to select only eQTLs where the variant resided within a TF motif within a promoter region (described in Note S1) *and* where the eGene was the gene adjacent to (and associated with) the promoter. Genotypes for NA12878 (corresponding to the GM12878 cell line) and K562 were downloaded on 06/10/2020. The Platinum Genomes genotype for NA12878 was obtained from `https://www.illumina.com/platinumgenomes.html` and the K562 genotype was obtained from ENCODE `https://www.encodeproject.org/files/ENCFF538YDL/` derived from a study by Zhou *et al.* (2019) (Zhou et al., 2019). Using the eQTL variants within motifs, we selected those variants where at

least one of the cell lines (K562 or GM12878) had at least one alternate allele of the eQTL variant. QBiC (Martin et al., 2019) was then run on these eQTLs, using hg19 as a reference genome. Significant QBiC disruptive effects of variants on TF binding were defined using the following criteria: (1) The predicted change in TF binding is negative indicating it alters a "canonical" TF site, and, (2) if the TF binding model was trained on human protein binding microarray (PBM) data the disruption is considered significant at a QBiC default p-value of 1×10^{-4}; if the TF binding model was trained on PBM data from a different species, the disruption is considered significant at a more stringent p-value of 1×10^{-20}. If the above two criteria were met, we assigned the variant a value of $q_{sij} = 1$, and 0 otherwise.

We elected to use only on negative TF binding effects (a negative QBiC value) in EGRET. This decision was motivated by two considerations. First, previous work (see Supplemental Figs. 2A-B in (Glass et al., 2015)) had shown that the message passing approach used in EGRET is robust to the removal of other unrelated TF-gene edges, suggesting that the overall network model should be robust to including only negative effects while allowing us to identify network differences between genotypes. This is supported by the observation that the predictive value of the entire GRN (based on ChIP-seq binding) is relatively robust, as described in Note S8. Second, identifying SNPs that have positive effects, which could create new binding motifs, would require a motif scan for each genotype (or the testing of each variant position in the genome) to identify new motifs created by a individual's unique variants; such motif scans are computationally expensive.

Note S3 Prior modification

When running EGRET, a genotype-specific prior ("EGRET prior") is constructed for each individual. For each SNP within a given individual, the alternate allele count of the individual is calculated. For each eQTL variant s in promoter region of gene j within a motif for TF i, three attributes are assigned: (1) the alternate allele count of the individual at that location $A_{s_{ij}}$ (2) the beta value of the eQTL $\beta_{s_{ij}}$ and (3) the QBiC effect of the SNP $q_{s_{ij}}$ on the binding of the TF corresponding to the motif in which the variant resides (only significant negative QBiC values are used). The effect of a SNP on TF binding in the given individual is then defined as the product $|q_{s_{ij}} A_{s_{ij}} \beta_{s_{ij}}|$. Modifier weights to the reference motif prior are then calculated by aggregating these effects per TF-gene pair, allowing for the fact that a gene might have more than one variant in its promoter region affecting the binding of a particular TF. The genotype-specific prior edge weight E_{ij} for TF i and gene j is thus defined as

$$E_{ij} = M_{ij} - \sum_{s} |q_{s_{ij}} A_{s_{ij}} \beta_{s_{ij}}|$$

where M_{ij} is the reference motif prior defined above in Note S1.

The small number of modified edges (1,520 for GM12878 and 1,182 for K562 out of a total of 39,690,052 possible edges) is a result of the stringent, successive filters we set for a TF-to-gene regulatory relationship (edge) to be disrupted. For an edge ij (TF motif i within the promoter of gene j to be disrupted), (1) the TF motif i needs to contain a SNP for which the individual has the alternate allele; (2) this variant needs to be a significant eQTL affecting the expression of gene j; and (3) this variant needs to be

predicted by QBiC to have a significant NEGATIVE effect on the binding of TF i at that specific genomic location. These three requirements, when required simultaneously, result in a relatively small amount of edges to be disrupted. The advantage of this approach is EGRET's ability to identify an individual's genetic variants that disrupt their TF regulatory network through a hypothesized—and thus falsifiable—mechanism (disruption of TF binding and regulation of the target gene).While these are promising results for identifying genotype-derived regulatory differences, we acknowledge that GM12878 and K562 cells, while both derived from blood, are not two genotypes of identical cell types, and that this is a limitation in the validation analysis.

Note S4 Gene expression and PPI data

Gene expression data as TPMs (transcripts per million) for lymphoblastoid cell lines (LCLs) from The Genotype-Tisse Expression Project (GTEx) version 7 (Lonsdale et al., 2013), was downloaded from https://gtexportal.org/home/datasets on 06/10/2020. The expression matrix was pruned to keep only genes that had non-zero expression values in at least 50 samples. The protein-protein interaction network is the same as used in (Sonawane et al., 2017). Briefly, human protein-protein interactions of transcription factors from StringDb version 10 (https://string-db.org) were used to construct a PPI network. StringDb PPI scores range from 0 to 1, and are an indicator of the confidence of the interaction. We filtered this PPI network to keep only proteins whose corresponding genes met the same expression requirements described above (non-zero expression values in at least 50 samples). When included in message passing, the PPI interaction scores are not thresholded, edge weights are included in the network overlap measures of message passing.

Thus, when selecting the set of genes and TFs to be included in the GRN, we removed any TFs or genes that did not have reasonable evidence of expression, where we defined "reasonable evidence of expression" as having non-zero values in $\geq$50 samples. Gene ID mapping from TF gene names to ensembl IDs was done using the mapping downloaded from ftp://ftp.ensembl.org/pub/grch37/current/gtf/homo_sapiens/Homo_sapiens.GRCh37.87.chr.gtf.gz on 06/10/2020.

Note S5 Message Passing Parameters

The refinement of E through message passing has three main practical advantages. First, and perhaps most importantly, edge weights are updated to reflect context-specificity from the gene-gene co-expression data. We have found this to be extremely valuable when analyzing gene regulatory networks without genotype information (for example, see (Sonawane et al., 2017)). This context-specificity is also demonstrated in our analysis of the different cell-type-specific EGRET networks from the same Yoruba individual. Second, message passing makes all edges (modified and unmodified) comparable, which allows users to calculate higher-level network metrics (node degree, network clusters/communities, etc.) which rely on this comparability. Third, message passing of E with C (gene-gene correlation matrix) and P (PPI matrix) provides a slight improvement to the overall network structure (about a 1.5% in-

crease AUC accuracy for predicting ChIP-seq binding in our particular example for GM12878). When running the message passing step using the pandaR package, the following parameters were used:

remove.missing.ppi = TRUE, remove.missing.motif = TRUE, remove.missing.genes = TRUE. These parameters ensure that the set of TFs is defined by those in the motif-gene prior, and that the set of genes is defined as the intersection of those in the motif-gene prior and the gene expression matrix.

Note S6 Computational Requirements

EGRET can feasibly be run on thousands of individuals, provided the user has access a compute cluster or cloud computing like AWS/Google Cloud. Table S3 shows computational requirements from the GM12878 genotype benchmark run on a single m5n.12xlarge node (48 CPUs, 192 GiB memory) on AWS. One can see that 6 cores were used, and peak memory was approximately 78 GiB. The job took around 1.25 hours. If one were to compute 1,000 EGRET networks of similar size, this would be expected to take around $1.25 \times 1,000 = 1,250$ hours. If one had access to 30 such nodes (a reasonable expectation - the Longleaf cluster at UNC Chapel Hill contains 30 “big data nodes” which would meet these requirements) would on average bring the wall time down to 41.67 hours, (just over 1.5 days). Detailed outputs from the `time` utility can be seen below.

Resource usage for pre-processing:

```
Output created: preprocess_finalEGRET_v1_timing.nb.html
    Command being timed: "Rscript -e rmarkdown::render('preprocess_finalEGRET_v1_timing.Rmd')
    User time (seconds): 8742.26
    System time (seconds): 112.93
    Percent of CPU this job got: 187%
    Elapsed (wall clock) time (h:mm:ss or m:ss): 1:18:52
    Average shared text size (kbytes): 0
    Average unshared data size (kbytes): 0
    Average stack size (kbytes): 0
    Average total size (kbytes): 0
    Maximum resident set size (kbytes): 25848184
    Average resident set size (kbytes): 0
    Major (requiring I/O) page faults: 169
    Minor (reclaiming a frame) page faults: 44071704
    Voluntary context switches: 78153
    Involuntary context switches: 402945
    Swaps: 0
    File system inputs: 60544472
    File system outputs: 5482936
    Socket messages sent: 0
    Socket messages received: 0
```

```
Signals delivered: 0
Page size (bytes): 4096
Exit status: 0
```

Resource usage for running EGRET:

```
Output created: timing_final_runEgret_gm12878_allQBiCModels.nb.html
    Command being timed: "Rscript -e rmarkdown::render('timing_final_runEgret_gm12878_allQBiC
    User time (seconds): 19753.61
    System time (seconds): 7355.42
    Percent of CPU this job got: 604%
    Elapsed (wall clock) time (h:mm:ss or m:ss): 1:14:42
    Average shared text size (kbytes): 0
    Average unshared data size (kbytes): 0
    Average stack size (kbytes): 0
    Average total size (kbytes): 0
    Maximum resident set size (kbytes): 77884748
    Average resident set size (kbytes): 0
    Major (requiring I/O) page faults: 102
    Minor (reclaiming a frame) page faults: 1145566516
    Voluntary context switches: 19825
    Involuntary context switches: 275841212
    Swaps: 0
    File system inputs: 33080
    File system outputs: 3574536
    Socket messages sent: 0
    Socket messages received: 0
    Signals delivered: 0
    Page size (bytes): 4096
    Exit status: 0
```

Note S7 Comparison of EGRET networks from two cell line genotypes

Note S7.1 ChIP-seq regulatory network

ChIP-seq data from ReMap2018 for GM12878 and K562 (Chèneby et al., 2018) (hg19 reference genome) was downloaded from http://pedagogix-tagc.univ-mrs.fr/remap/index.php?page=download on 06/12/2020. This consisted of genomic ranges in BED format corresponding to the identified binding positions of several transcription factors (110 TFs for GM12878 and 204 TFs for K562). From this ChIP-seq data, TF binding sites within the promoter regions of genes were selected in the same manner as as

the motif regions, described above. This resulted in two validation networks V, one for each cell line, where

$$V_{ij} = \begin{cases} 1 & \text{if ChIP-seq range of TF } i \text{ overlaps with promoter region of gene } j \\ 0 & \text{otherwise} \end{cases}$$

The subset of TFs for which ChIP-seq data was available in a given genotype (GM12878 or K562) were then used for subsequent analysis involving comparison of EGRET networks with ChIP-seq networks.

Note S7.2 Improving prediction of TF binding

The top edges with the highest disruption scores $d^{(E)}_{x_{ij}}$ were selected from the EGRET GM12878 and K562 networks, using a selection of different $d^{(E)}_{x_{ij}}$ cutoffs to define the top set of edges (Tables S5 and S6). Using the EGRET edge score as the predictor variable and the edges from the gold standard ChIP-seq GRN V as the ground truth, we calculated performance metrics, namely the area under the receiver-operator characteristic (AU-ROC) and the area under the precision-recall (AU-PR) curve for edges with the top disruption scores $d^{(E)}_{x_{ij}}$; this was repeated for different thresholds of the edge disruption score. To compare the EGRET edge weights with those from the genotype-agnostic network, we calculated the significance between the differences of the AUCs using the Delong test for comparing AUCs (Tables S5 and S6). In both GM12878 and K562, the genotype-specific edges significantly improved the prediction of TF binding on variant-impacted edges. An optimal threshold of $d^{(E)}_{x_{ij}} \geq 0.35$ was identified for the isolation of variant impacted edges, as this was the threshold at which ChIP-seq TF binding predictions improved significantly for both GM12878 and K562. AU-ROCs and AU-PRs were calculated using the precrec (Saito and Rehmsmeier, 2017) and pROC (Robin et al., 2011) R packages.

Note S7.3 Allele-specific expression

Allele-specific expression (ASE) data using the BiT-STARR-seq method in LCLs (Kalita et al., 2018) was downloaded from `https://genome.cshlp.org/content/suppl/2018/10/17/gr.237354.118.DC1/Supplemental_Table_S1_.txt` on 09/01/2020. This data contained all variants tested for an ASE association, and these variants were mapped to TF motif regions in the promoter regions of genes, in the same manner as described above. Each gene j was then assigned *gene regulatory difference score* $R^{(G)}_j$ defined as:

$$R^{(G)}_j = \sum_i R^{(E)}_{ij}.$$

This score agglomerates the *edge regulatory difference scores* per gene, providing a metric quantifying the total extent to which a gene's promoter region is differentially disrupted between the two cell lines. We then used Fisher's exact test to determine whether genes with a high regulatory difference score $R^{(G)}_j$ between the two genotypes K562 and GM12878 were enriched for genes having a significant (FDR ≤ 0.1) ASE variant within a motif in their promoter region. "High" regulatory difference scores were considered to be those in to top 10%.

Note S7.4 Chromatin accessibility QTLs

Chromatin accessibility QTLs (caQTLs) determined in lymphoblastoid cell lines (LCLs) (Banovich et al., 2018) were downloaded from http://eqtl.uchicago.edu/yri_ipsc/cht_results_full_LCL.txt on 09/08/2020 (Banovich et al., 2018). This data set contained all variants tested for a caQTL association. We mapped these variants to TF motif regions in the promoters of genes (described above in Note S2). We again used the regulatory difference scores $R_j^{(G)}$ to test whether genes with a high regulatory difference score $R_j^{(G)}$ between the two genotypes K562 and GM12878 were enriched for genes having a significant (FDR ≤ 0.1) caQTL variant within a motif in their promoter region, using Fisher's exact test in a similar manner as described above.

Note S8 Sensitivity analysis

We investigated the sensitivity of EGRET to the two most variable parameters - the significance threshold for the motif prior, and the significance threshold for the eQTLs. As can be seen in Figure S8, increasing the stringency of the motif prior significance threshold negatively affects the accuracy of the global network when validating against the gold-standard ChIP-seq network from GM12878. Thus, we do not recommend decreasing the motif prior threshold below the default for FIMO, which is 1e-4. The eQTL parameter is one which we feel can be more safely altered by the user depending on whether sensitivity or specificity is of most importance (Figure S9). Decreasing the p-value cutoff (and thus increasing the stringency of the threshold) has no impact on the accuracy of global network structure (Figure S9A), but can provide some small increases in accuracy of the variant-disrupted edges (Figure S9B). However, this is at the cost of significantly lowering the sample size of edited edges (Figure S9C).

Note S9 Population study of 119 individuals across 3 cell types

Note S9.1 Network Construction

Gene expression and eQTL data for a population of lymphoblastoid cell lines (LCL), induced pluripotent stem cells (iPSCs) and cardiomyocytes (CMs) that were differentiated from the induced pluripotent stem cells derived the study by Banovich *et al.* (Banovich et al., 2018) and Li *et al.* (Li et al., 2016), as well as the corresponding genotypes of 119 Yoruba individuals were downloaded on 06/17/2020. Expression data and eQTLs for LCLs, as well as eQTLs for iPSCs and iPSC-CMs were downloaded from http://eqtl.uchicago.edu/ whereas gene expression data for iPSCs and CMs were obtained through the Gene Expression Omnibus (GEO) from https://www.ncbi.nlm.nih.gov/geo/query/acc.cgi?acc=GSE107654. For each cell type, significant eQTLs ($p \leq 1 \times 10^{-5}$) for genes where the SNP resided within a TF motif within the promoter region of a gene ([-750,+250] around a TSS) were selected.

For each cell type, SNPs in the population of 119 Yoruba individuals that also were selected as eQTLs in the respective cell type were then isolated, and QBiC was run on this set of SNPs, per cell type, as in Note S3.

LCL and iPSC expression data were already preprocessed through WASP and normalized by standardizing by gene and quantile normalizing by individual, a method developed and used in (Degner et al., 2012). The CM expression was not yet normalized, and we followed the process detailed in the series matrix files from GEO https://www.ncbi.nlm.nih.gov/geo/query/acc.cgi?acc=GSE107654 in order to process the CM expression data in the same manner. This involved scaling each gene by mean centering and dividing by the standard deviation, followed by quantile-normalizing the individuals using the normalize.quantiles function in the preprocessCore R package (Bolstad et al., 2003). QBiC (Martin et al., 2019) was then run on the eQTLs to predict the effect these SNPs had on the binding of TFs using the full set of TF binding models in QBiC, and using hg19 as a reference genome.

EGRET was then run for each genotype in each cell type (a total of $119 \times 3 = 357$ EGRET runs). In addition, message passing was performed using the co-expression network, PPI network, and the reference motif prior (which involves no genotype information) to construct a "genotype agnostic" baseline GRN for each cell type. Message passing was performed using the pandaR R package (Glass et al., 2013) and run in parallel using GNU Parallel (Tange, 2011).

Note S9.2 TF disruption scores

Edge disruption scores $d^{(E)}_{x_{ij}}$ were calculated for each edge in each individual network for each cell type, and thresholded at a value of 0.35. Subsequently, a TF disruption score $d^{(TF)}_{x_i}$ was calculated for each TF as

$$d^{(TF)}_{x_i} = \sum_j d^{(E)}_{x_{ij}}.$$

A scaled TF disruption score $d^{(TF)'}_{x_i}$ for a TF within in an individual and cell type was then calculated by subtracting the mean TF disruption score for that individual/cell type and dividing by the standard deviation. Disease-associated genes for coronary artery disease (CAD) and Crohn's disease (CD) were obtained from the GWAS catalog at https://www.ebi.ac.uk/gwas/api/search/downloads/full on 06/30/2020 (Buniello et al., 2019). See Tables S7 and S8 for a complete list of citations for the individual GWAS studies from which summary statistics were used.

Note S9.3 Differential modularity with ALPACA

For each individual, we used ALPACA (Padi and Quackenbush, 2018) to compare the modularity of the individual's genotype-specific EGRET GRN with the baseline GRN, resulting in a score for each node representing the contribution of that node to the differential modularity. These scores were then quantile normalized per individual per cell type. Following that, scores were normalized by gene, first by mean-centering and then by scaling to standard deviation of one.

Note S9.4 Functional Enrichment

Note S9.4.1 Functional enrichment in genes with high DM scores in individual 18.

Gene ontology enrichment of genes ranked by differential modularity in individual 18 was performed using GORILLA (Eden et al., 2009) on the web server available at http://cbl-gorilla.cs.technion.ac.il/ using the "Single ranked list of genes" option and a p-value threshold of 10^{-3}.

Note S9.4.2 Functional enrichment in different communities in individual 18

Using the g:Profiler R package (Raudvere et al., 2019) we determined functional enrichment of terms from GO biological process, KEGG and Reactome ontologies. Using the network communities derived from ALPACA, we show that different communities exist within the EGRET network of an individual 18. These communities are enriched for different functional processes (Figures S19, S20, and S21). It is interesting to note that *CSRP1*, the known smooth muscle associated with the bundling of actin filaments that contributes to the high TF disruption score for ERG in individual 18 is located within community 2, which is enriched for cytoskeleton-related functions. This illustrates how that the global network structure is useful to provide context for interpretation for where mutations and disrupted regulatory edges reside.

Supplementary Figures

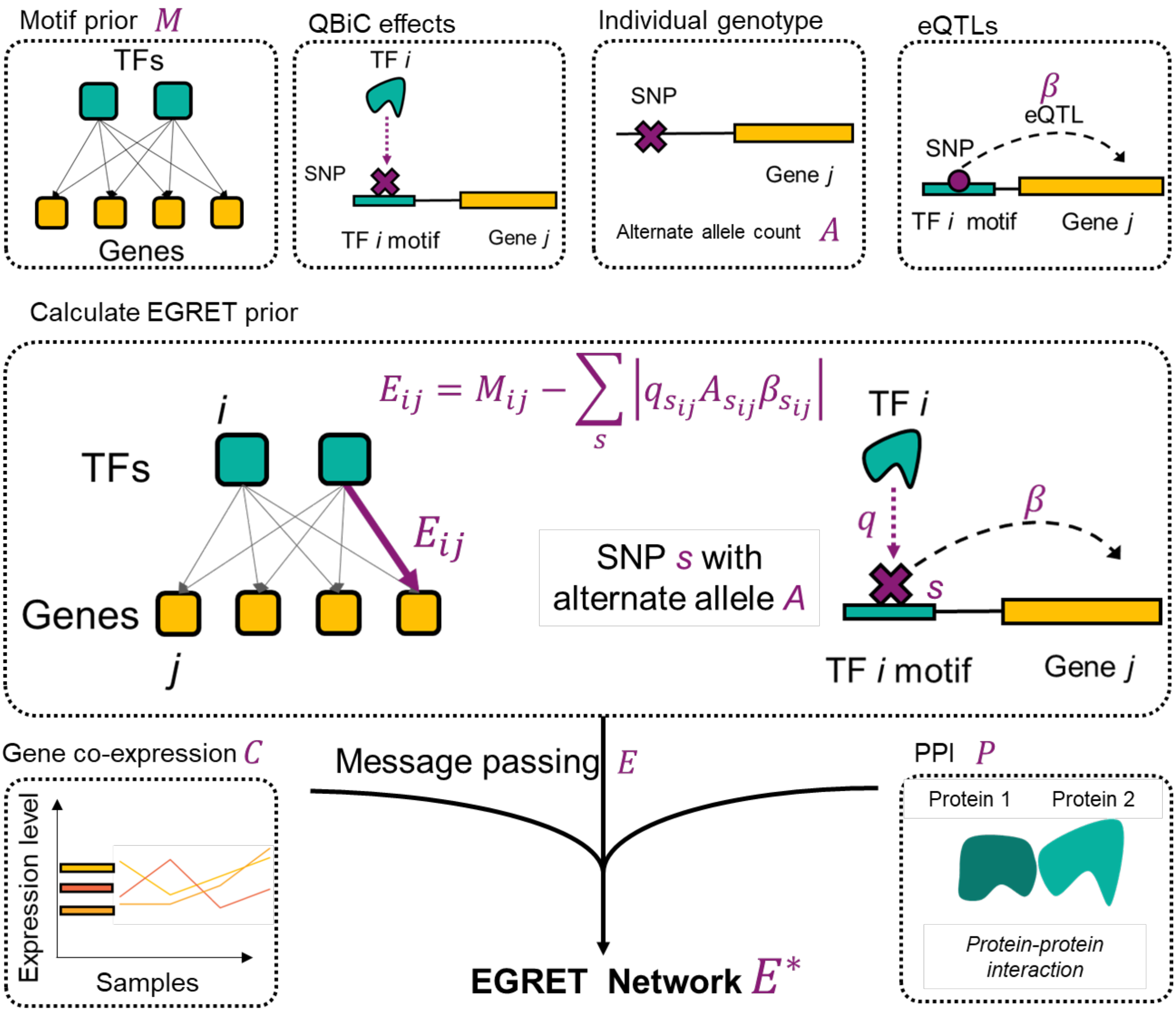

Figure S1: **Diagram illustrating the process and datatypes required for EGRET network construction.** EGRET begins with a reference motif prior representing the presence/absence of TF motifs in the promoter regions of genes. This is then modified by the individual's genetic mutations, penalizing motif-gene edges in which there exists a variant within the TF motif for which the individual has the alternate allele (A), the variant is an eQTL for the adjacent gene (β) and the variant is predicted through QBiC to disrupt TF binding at that location (q). These prior edges are then penalized by the absolute value of the product of the alternate allele count, the QBiC effect, and the eQTL beta value. Message passing then integrates the co-expression network (C) and PPI network (P) with the EGRET prior (E), resulting in a final genotype-specific GRN per individual (E^*).

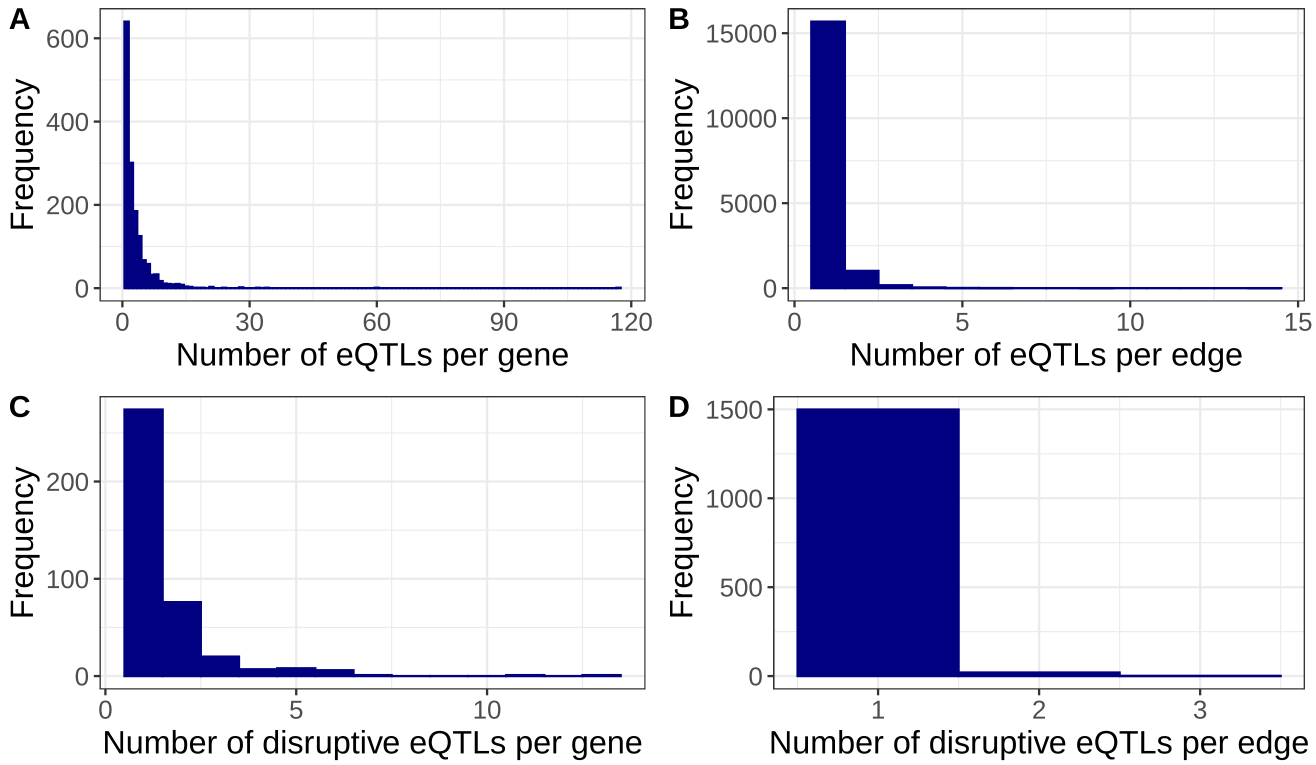

Figure S2: **eQTLs per gene/edge.** (A) Distribution of number of eQTLs which fall within the promoter regions of genes (i.e., fall within any TF motif within the promoter region of a given gene); (B) Distribution of number of eQTLs which fall within a particular TF's motif within a particular gene's promoter; (C) Distribution of number of disruptive (significant negative QBiC effect - see Note S2) eQTLs which fall within the promoter regions of genes (i.e. fall within at least one TF motif within the promoter region of a given gene); (D) Distribution of number of disruptive (significant negative QBiC effect - see Note S2) eQTLs which fall within a particular TF's motif within a particular gene's promoter.

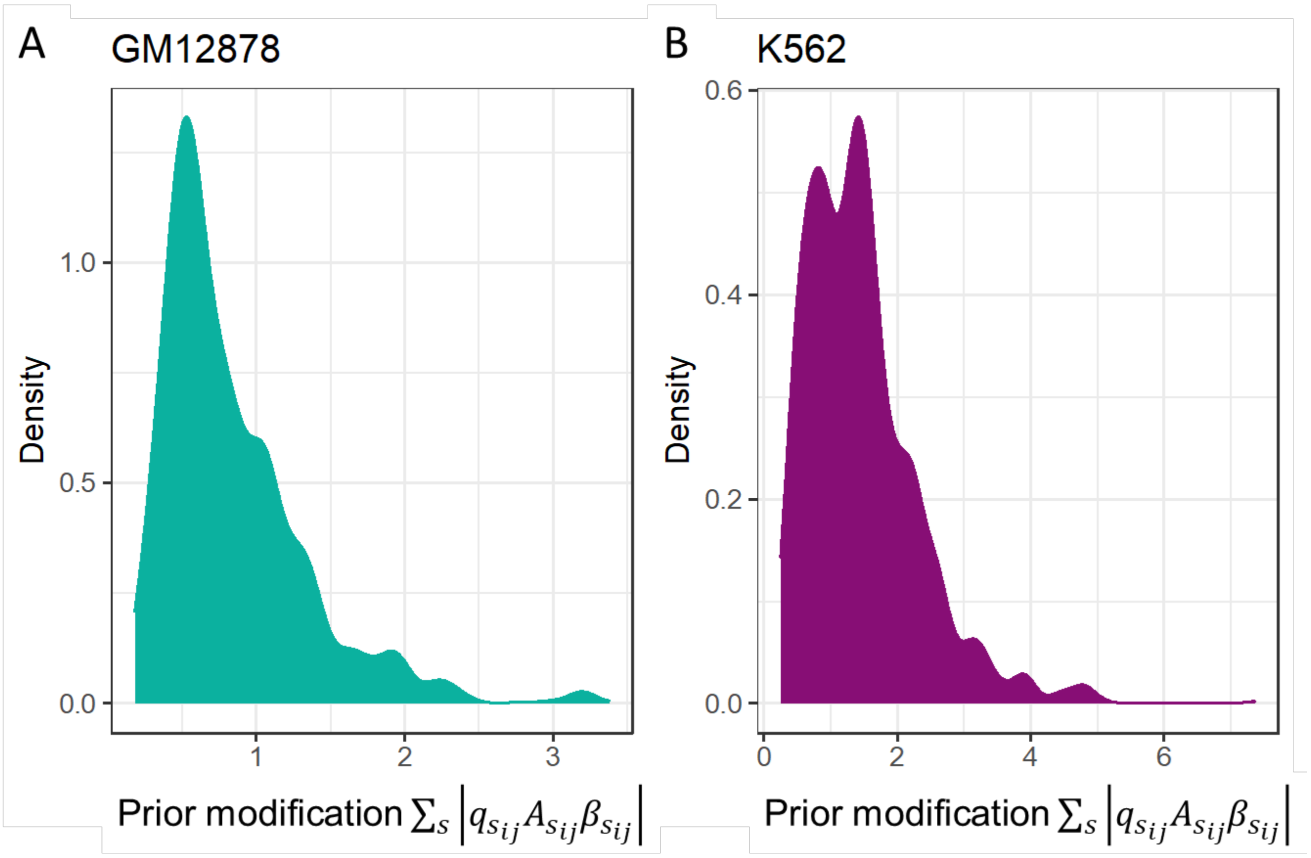

Figure S3: **Distribution of non-zero prior modifications** $\sum_s |q_{s_{ij}} A_{s_{ij}} \beta_{s_{ij}}|$ for (**A**) GM12878 and (**B**) K562.

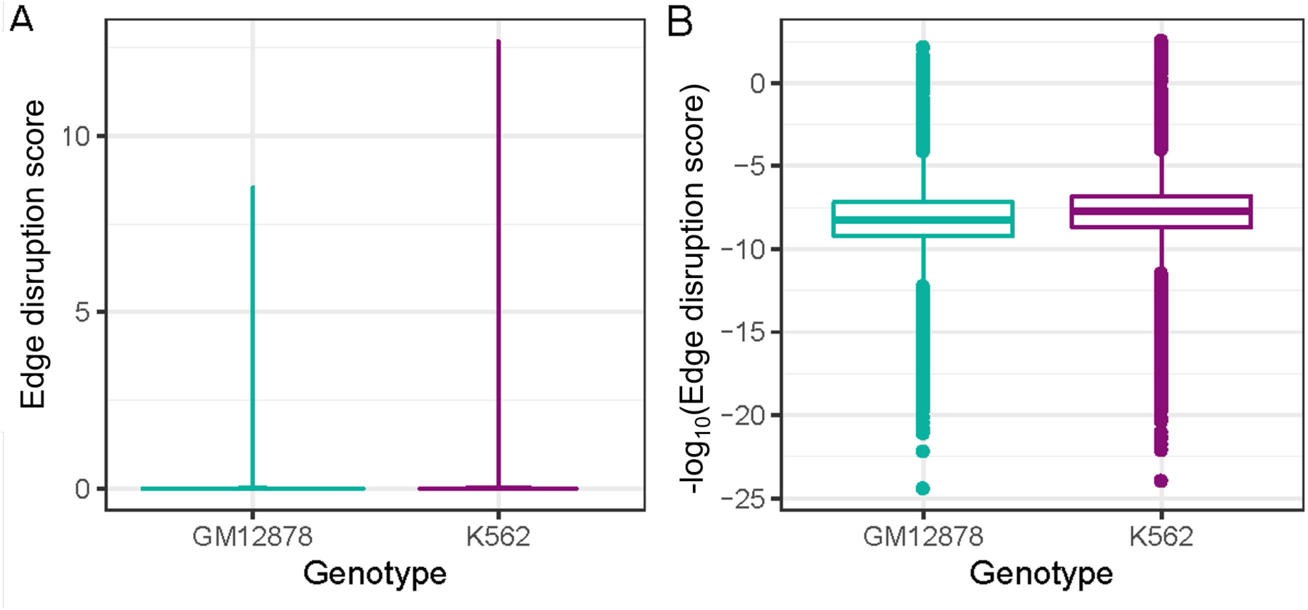

Figure S4: **Distribution of edge disruption scores** $d_{x_{ij}}^{(E)}$ **for GM12878 and K562.** (**A**) Violin plot of edge disruption scores. (**B**) Boxplot of $\log_{10}$ disruption scores.

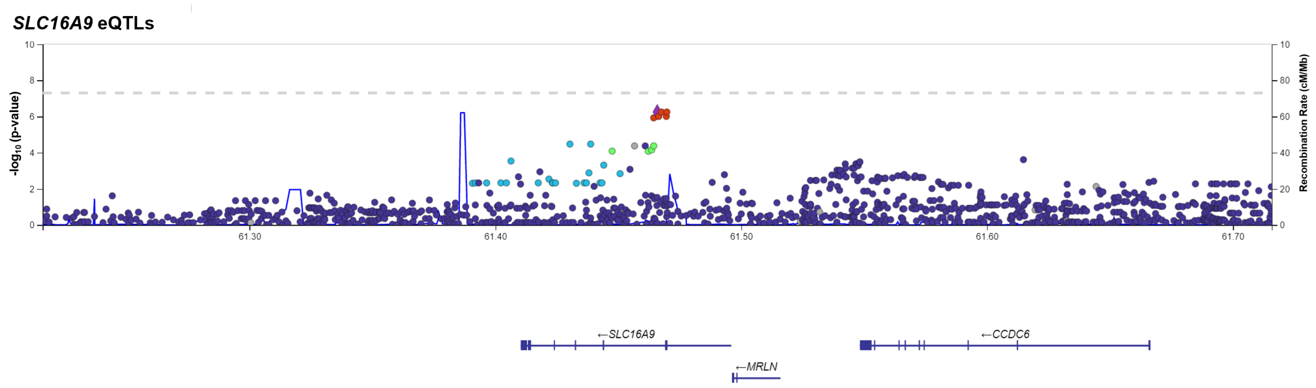

Figure S5: ***SLC16A9* region eQTLs.** LocusZoom plot (Boughton et al., 2021) of GTEx LCL eQTLs in the region of *SLC16A9*.

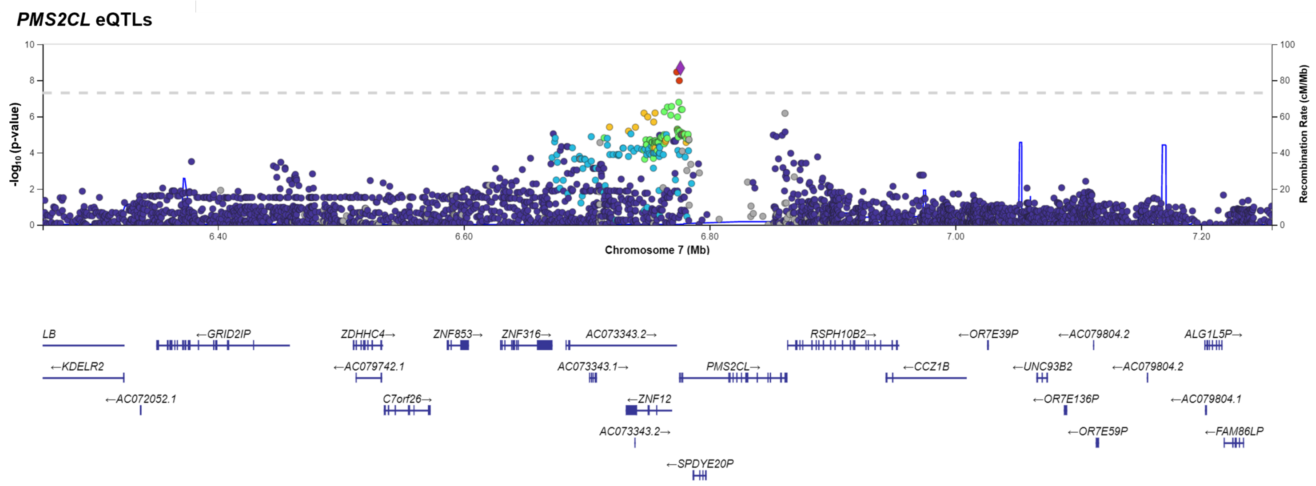

Figure S6: ***PMS2CL* region eQTLs.** LocusZoom plot (Boughton et al., 2021) of GTEx LCL eQTLs in the region of *PMS2CL*.

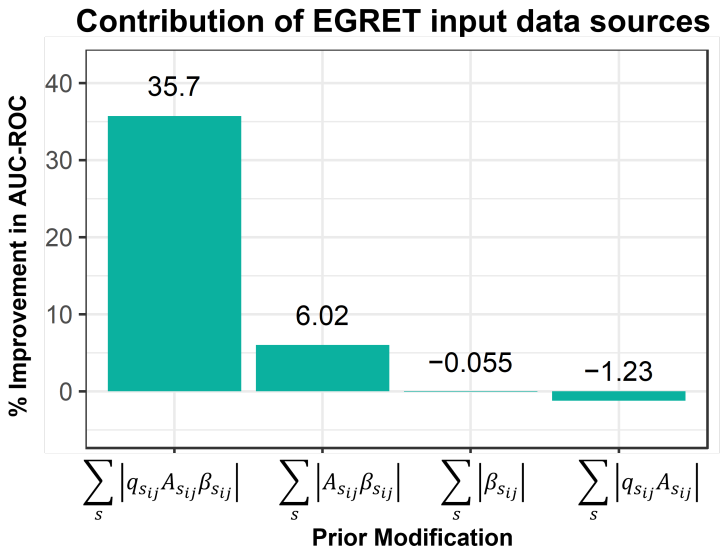

Figure S7: **Contribution of different data types to EGRET.** Percentage improvement in the prediction of the ChIP-seq regulatory network by the EGRET network E^* in GM12878, compared to that of the baseline network B^*. Each bar represents the AUC-ROC improvement when using a different combination of data types in the prior modification, for each SNP s with QBiC effect q, alternate allele count A and eQTL beta value β. Percentage improvement calculated as $(AUC_{E^*} - AUC_{B^*})/AUC_{B^*}$

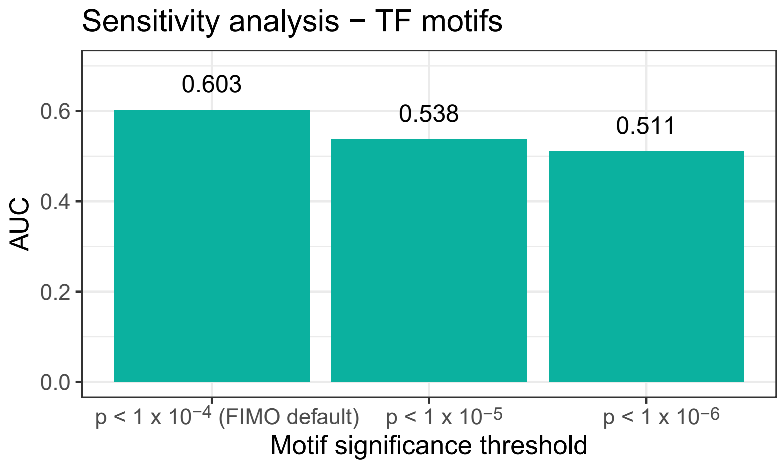

Figure S8: **Sensitivity analysis - motif calls.** Accuracy of the global network structure, validated against the gold-standard ChIP-seq network for GM12878, with different significance thresholds for the motif prior.

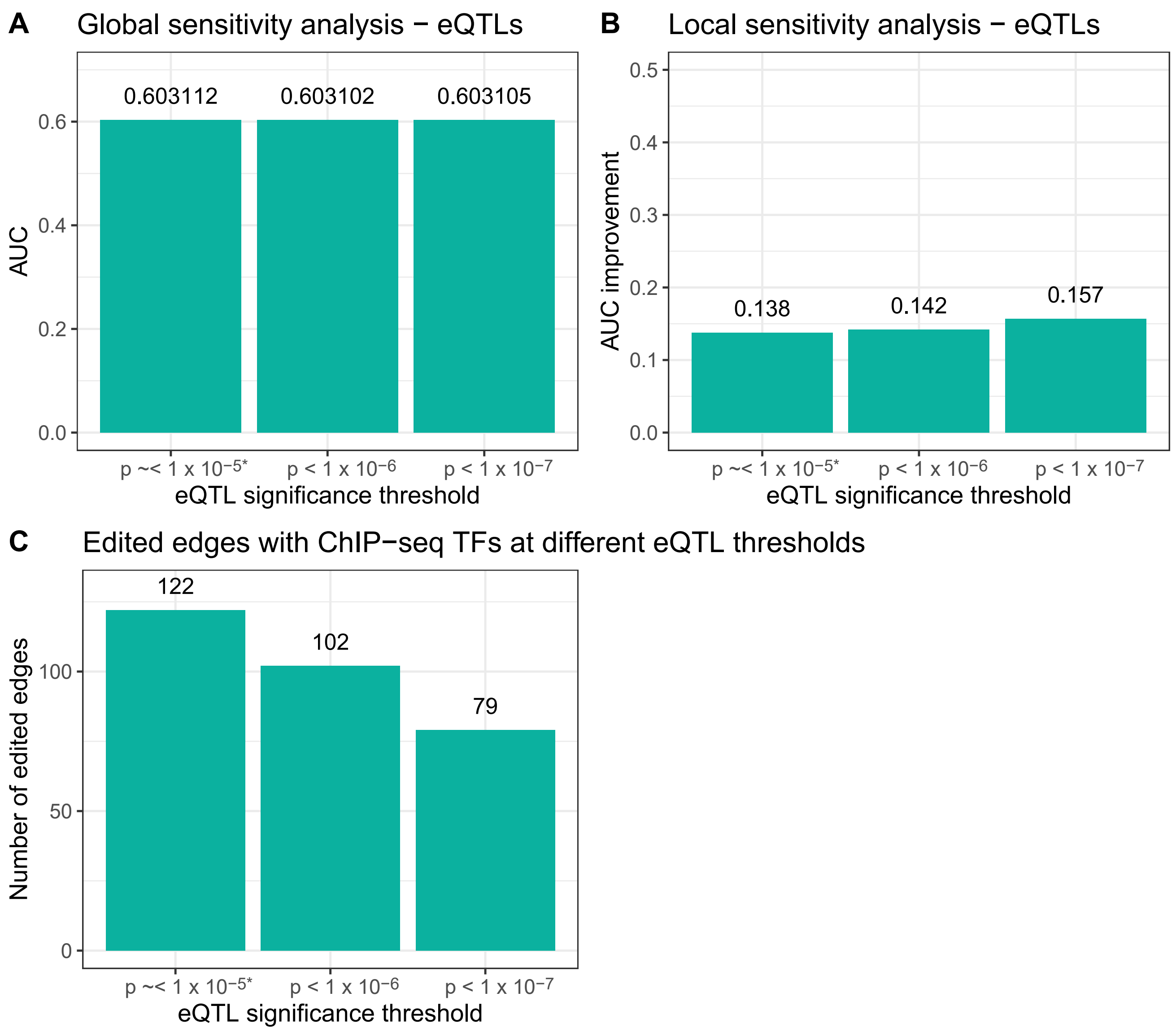

Figure S9: **Sensitivity analysis - eQTLs.**(A) Accuracy of the global EGRET network, validated against the gold-standard ChIP-seq network for GM12878, with different significance thresholds for calling eQTLs. (B) Accuracy of the variant disrupted edges, validated against the gold-standard ChIP-seq network for GM12878, with different significance thresholds for calling eQTLs. (C) Number of disrupted edges overlapping with ChIP-seq data at different eQTL thresholds. (*) See Note S2 for details on the default GTEx approach for determining the p-value cutoff.

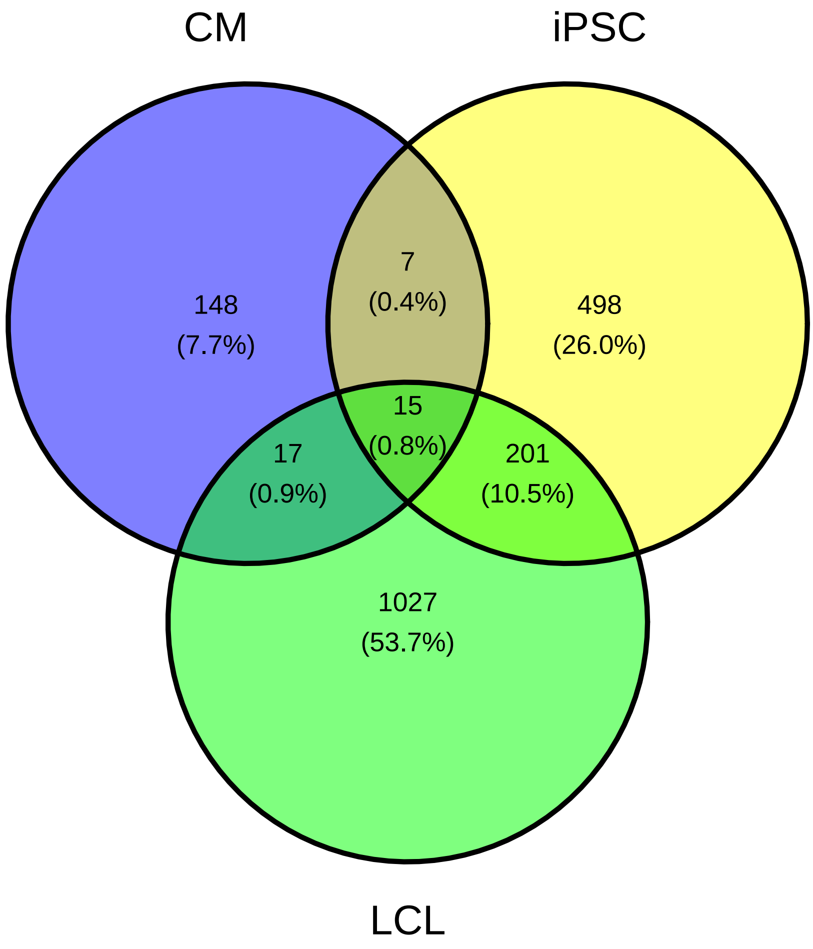

Figure S10: **Cell type eQTL overlap.** Venn diagram indicating the overlap of eQTLs between LCL, iPSC and CM cell types in the Banovich et al. dataset.

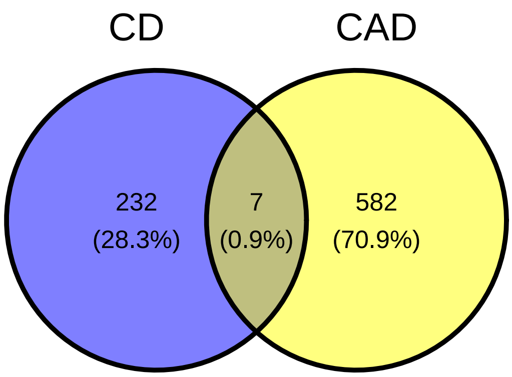

Figure S11: **CAD/CD TF overlap.** Venn diagram indicating the overlap between TFs associated with CAD and CD through GWAS.

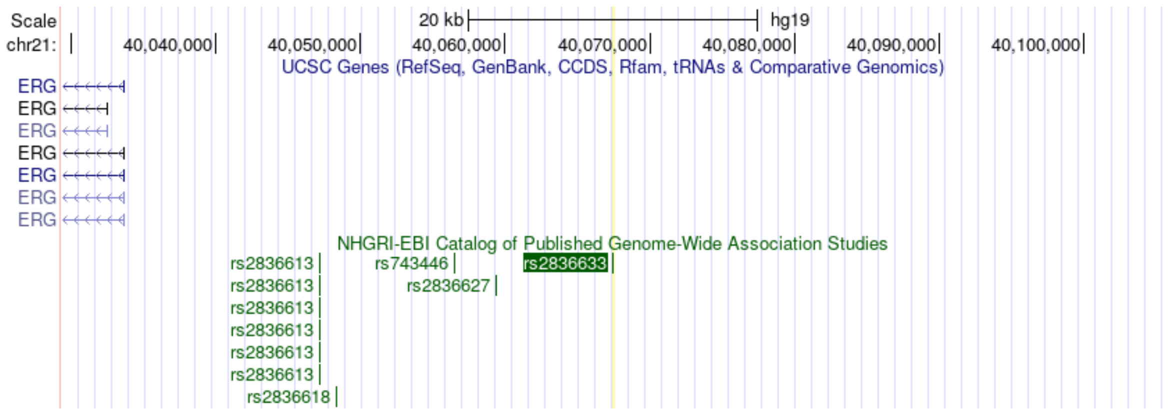

Figure S12: **GWAS SNP associated with CAD.** Position of SNP rs2836633 which is associated with CAD via a GWAS association.

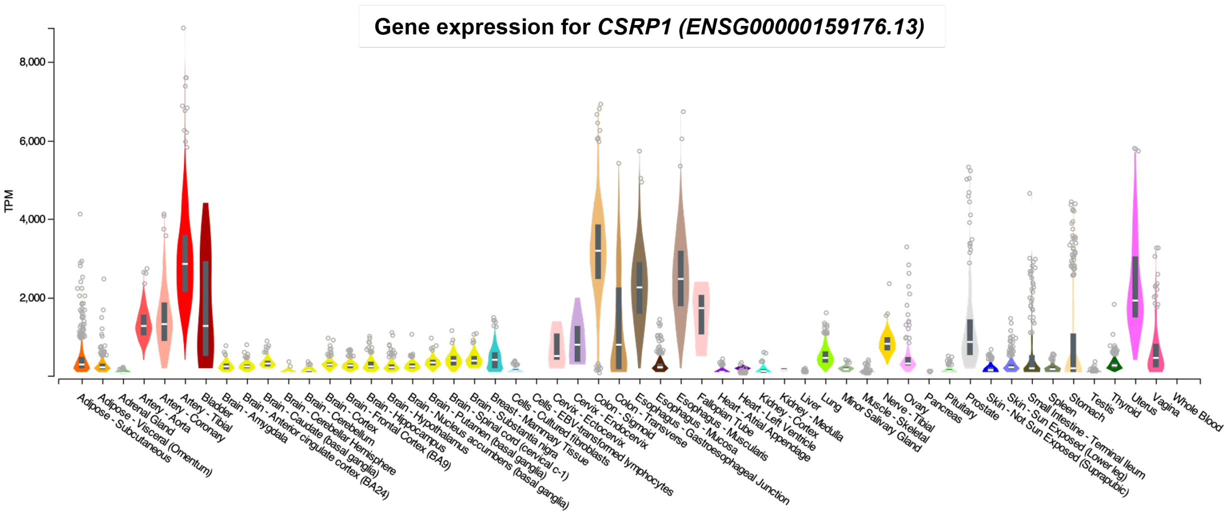

Figure S13: ***CSRP1* expression.** TPM expression level of *CSRP1* (*ENSG00000159176*) across all tissues available in GTEx. Plot obtained from the GTEx portal (Lonsdale et al., 2013).

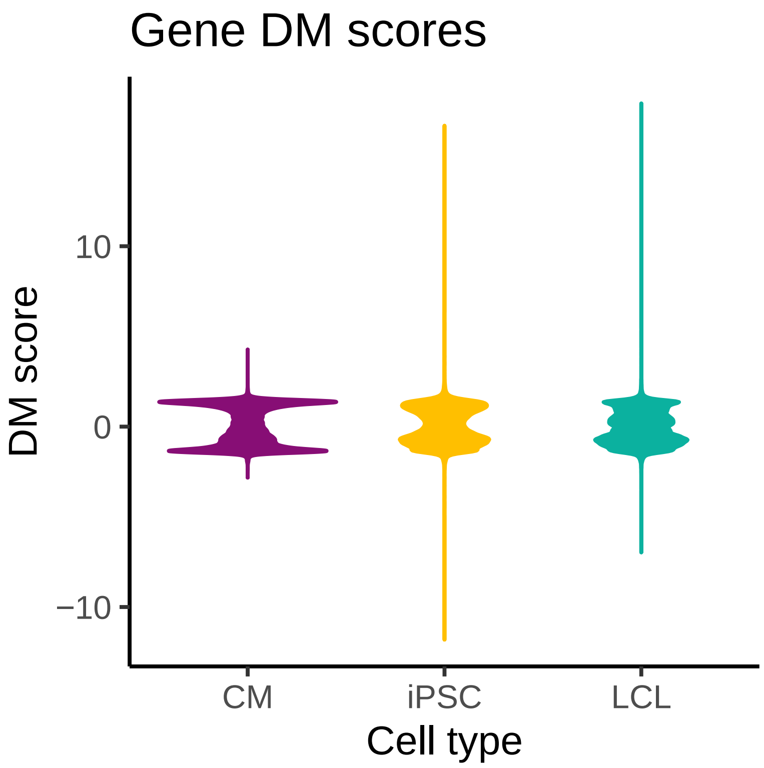

Figure S14: **Differential modularity scores.** Distributions of scaled differential modularity (DM) scores of genes in EGRET networks from 119 Yoruba individuals in three cell types.

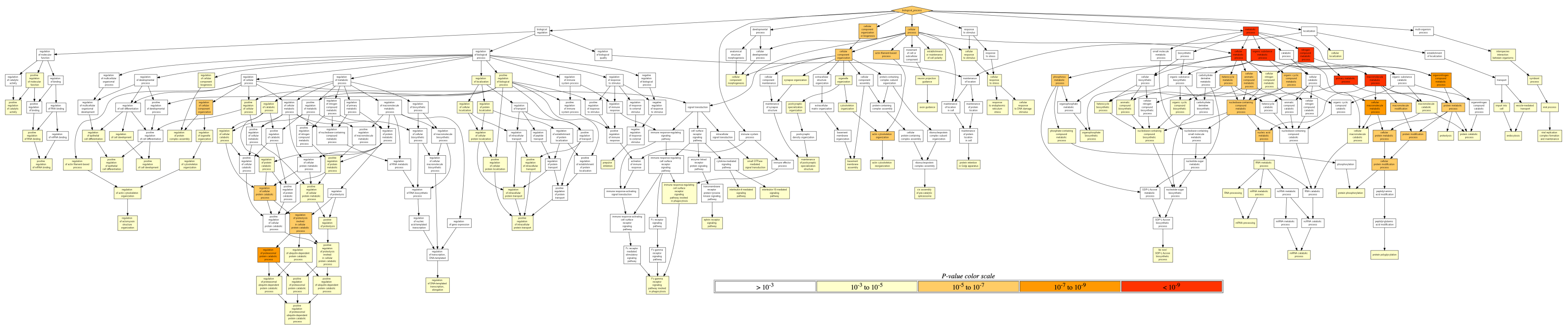

Figure S15: **Hierarchy of GO terms enriched in the genes with highest DM scores in individual 18 (genotype NA18523) in CMs, based on the mHG score test (Eden et al., 2009). Enrichment performed using GORILLA (Eden et al., 2009).** GO terms are colored according to the significance of the p-value.

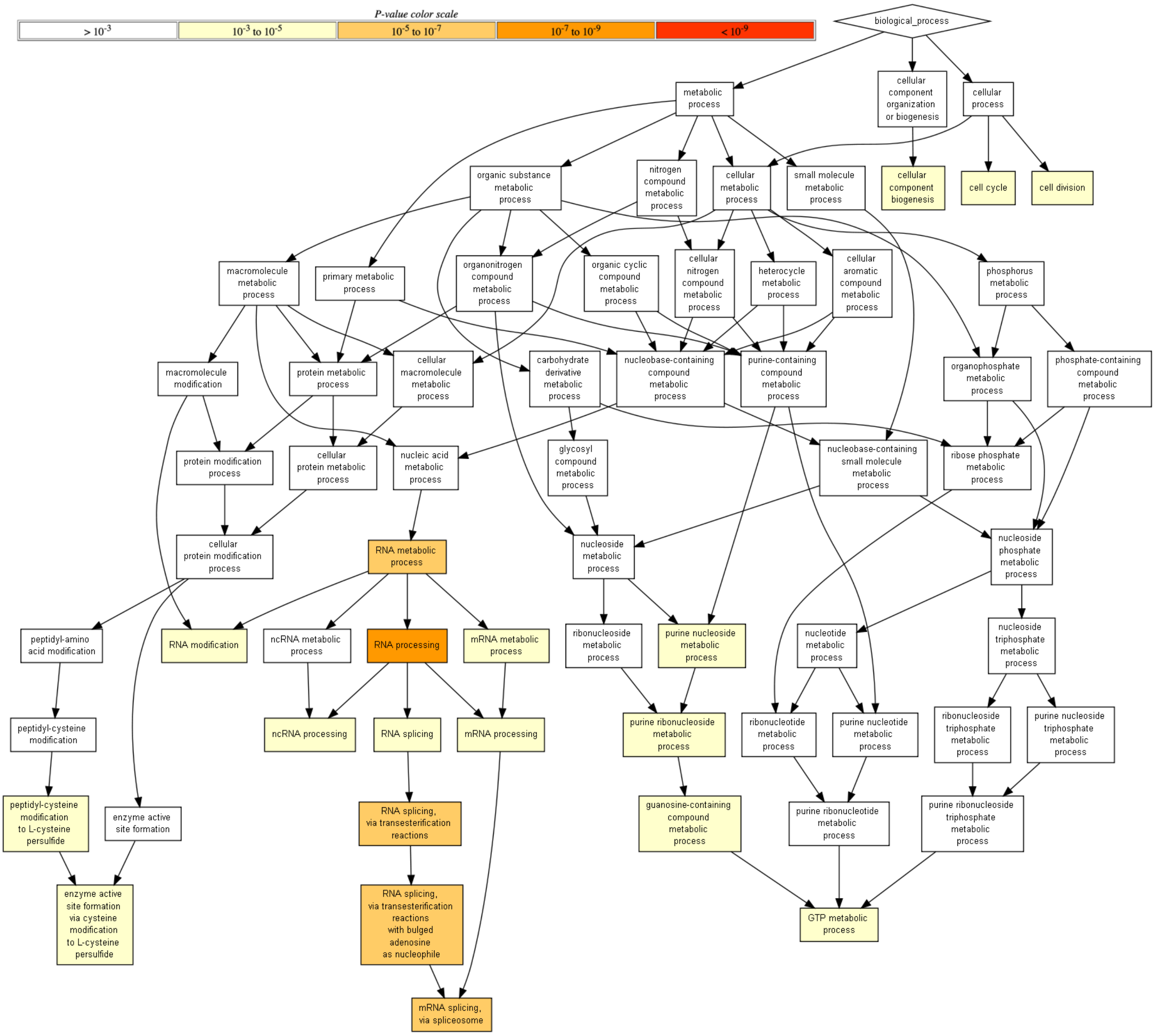

Figure S16: **Hierarchy of GO terms enriched in the genes with highest DM scores in individual 18 (genotype NA18523) in iPSCs, based on the mHG score test (Eden et al., 2009).** Enrichment performed using GORILLA (Eden et al., 2009). GO terms are colored according to the significance of the p-value.

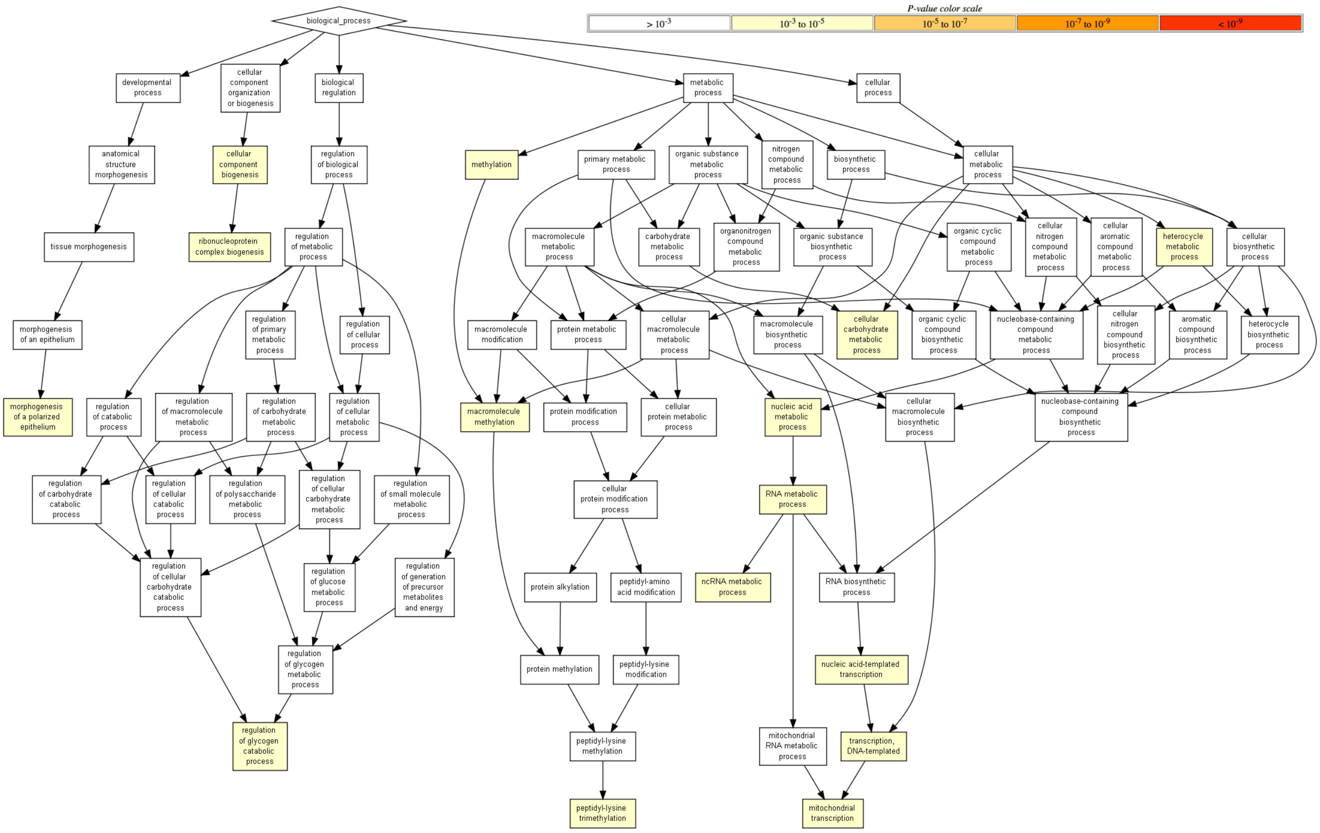

Figure S17: **Hierarchy of GO terms enriched in the genes with highest DM scores in individual 18 (genotype NA18523) in LCLs, based on the mHG score test (Eden et al., 2009).** Enrichment performed using GORILLA (Eden et al., 2009). GO terms are colored according to the significance of the p-value.

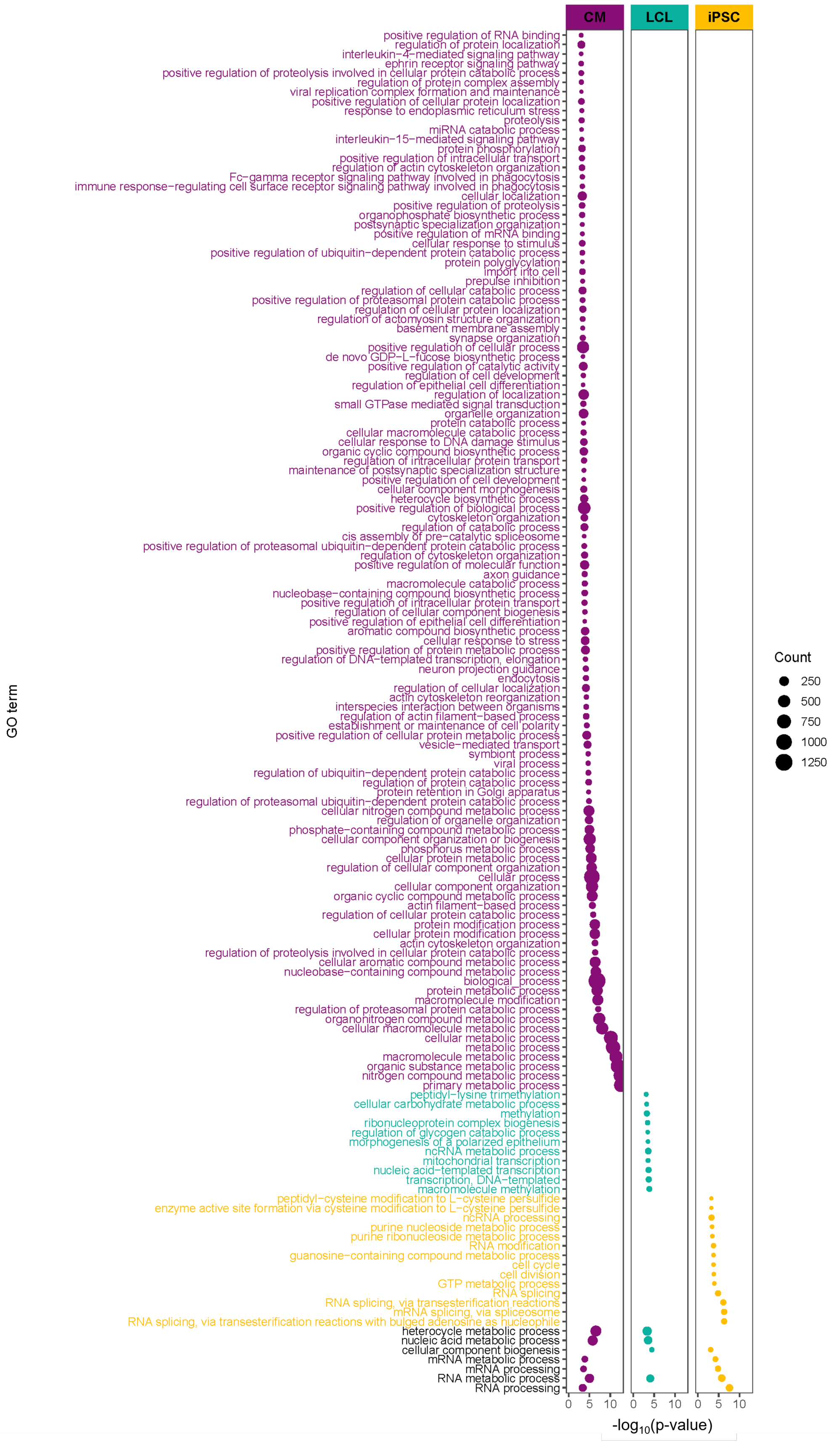

Figure S18: **GO terms enriched in genes with high DM scores in individual 18**, the individual with the highest TF disruption score for ERG. Point size corresponds to the the number of high-DM genes annotated with the corresponding GO term.

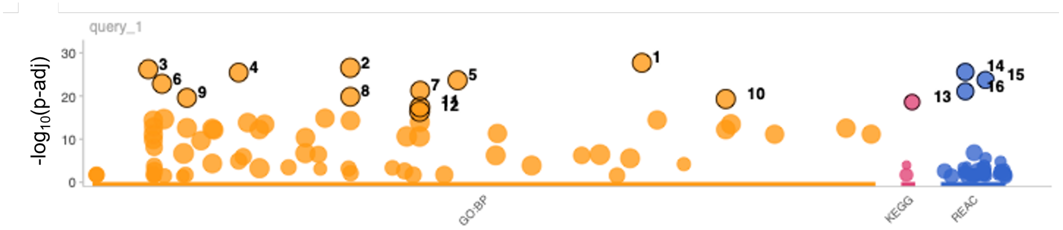

id	source	term_id	term_name	term_size		p_value
1	GO:BP	GO:0090304	nucleic acid metabolic process	4871		2.0e−28
2	GO:BP	GO:0034641	cellular nitrogen compound metabolic process	6089		3.0e−27
3	GO:BP	GO:0006139	nucleobase−containing compound metabolic process	5369		6.1e−27
4	GO:BP	GO:0016070	RNA metabolic process	4373		3.7e−26
5	GO:BP	GO:0046483	heterocycle metabolic process	5543		2.2e−24
6	GO:BP	GO:0006725	cellular aromatic compound metabolic process	5584		1.5e−23
7	GO:BP	GO:0044271	cellular nitrogen compound biosynthetic process	4657		5.7e−22
8	GO:BP	GO:0034645	cellular macromolecule biosynthetic process	4684		1.5e−20
9	GO:BP	GO:0009059	macromolecule biosynthetic process	4747		2.7e−20
10	GO:BP	GO:1901360	organic cyclic compound metabolic process	5824		5.0e−20
11	GO:BP	GO:0044260	cellular macromolecule metabolic process	7757		3.7e−18
12	GO:BP	GO:0044237	cellular metabolic process	10119		3.8e−17
13	KEGG	KEGG:05168	Herpes simplex virus 1 infection	464		2.6e−19
14	REAC	REAC:R−HSA−74160	Gene expression (Transcription)	1414		2.7e−26
15	REAC	REAC:R−HSA−73857	RNA Polymerase II Transcription	1282		1.9e−24
16	REAC	REAC:R−HSA−212436	Generic Transcription Pathway	1162		8.4e−22

g:Profiler (biit.cs.ut.ee/gprofiler)

Figure S19: **Functional enrichment in community 1.** Functional enrichment in community 1 of individual 18's EGRET network. Enrichment and visualization performed using the g:Profiler R package (Raudvere et al., 2019).

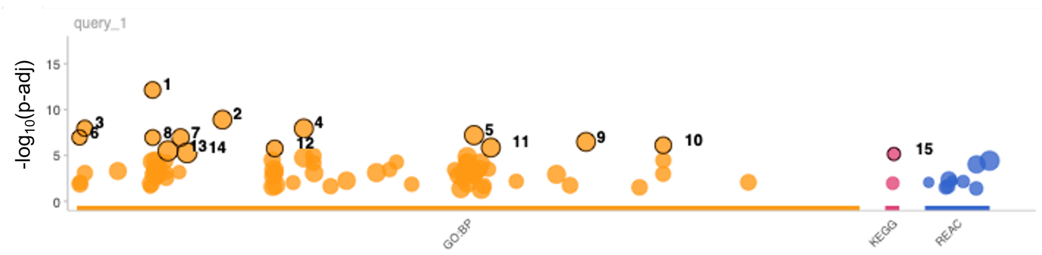

id	source	term_id	term_name	term_size		p_value
1	GO:BP	GO:0007010	cytoskeleton organization	1413		7.3e−13
2	GO:BP	GO:0016043	cellular component organization	6276		1.2e−09
3	GO:BP	GO:0000902	cell morphogenesis	1029		1.1e−08
4	GO:BP	GO:0032501	multicellular organismal process	7255		1.1e−08
5	GO:BP	GO:0050896	response to stimulus	8432		6.1e−08
6	GO:BP	GO:0000226	microtubule cytoskeleton organization	640		1.1e−07
7	GO:BP	GO:0009653	anatomical structure morphogenesis	2656		1.1e−07
8	GO:BP	GO:0007017	microtubule−based process	900		1.1e−07
9	GO:BP	GO:0071840	cellular component organization or biogenesis	6488		3.3e−07
10	GO:BP	GO:0120036	plasma membrane bounded cell projection organization	1559		7.7e−07
11	GO:BP	GO:0051716	cellular response to stimulus	7050		1.4e−06
12	GO:BP	GO:0030030	cell projection organization	1598		1.7e−06
13	GO:BP	GO:0008150	biological_process	16194		3.1e−06
14	GO:BP	GO:0009987	cellular process	15496		5.2e−06
15	KEGG	KEGG:04740	Olfactory transduction	216		6.8e−06

g:Profiler (biit.cs.ut.ee/gprofiler)

Figure S20: **Functional enrichment in community 2.** Functional enrichment in community 2 of individual 18's EGRET network. Enrichment and visualization performed using the g:Profiler R package (Raudvere et al., 2019).

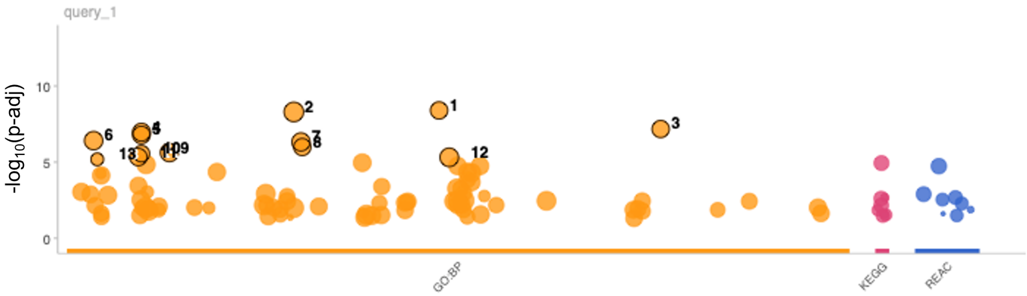

id	source	term_id	term_name	term_size		p_value
1	GO:BP	GO:0046903	secretion	1437		4.0e−09
2	GO:BP	GO:0032501	multicellular organismal process	7255		5.1e−09
3	GO:BP	GO:0140352	export from cell	1351		6.7e−08
4	GO:BP	GO:0006955	immune response	2111		1.1e−07
5	GO:BP	GO:0006952	defense response	1654		1.6e−07
6	GO:BP	GO:0002376	immune system process	2956		3.9e−07
7	GO:BP	GO:0032879	regulation of localization	2687		4.7e−07
8	GO:BP	GO:0032940	secretion by cell	1300		1.0e−06
9	GO:BP	GO:0009605	response to external stimulus	2763		2.4e−06
10	GO:BP	GO:0006954	inflammatory response	801		2.8e−06
11	GO:BP	GO:0006811	ion transport	1530		4.6e−06
12	GO:BP	GO:0048513	animal organ development	3494		4.9e−06
13	GO:BP	GO:0002526	acute inflammatory response	110		6.5e−06

g:Profiler (biit.cs.ut.ee/gprofiler)

Figure S21: **Functional enrichment in community 3.** Functional enrichment in community 3 of individual 18's EGRET network. Enrichment and visualization performed using the g:Profiler R package (Raudvere et al., 2019).

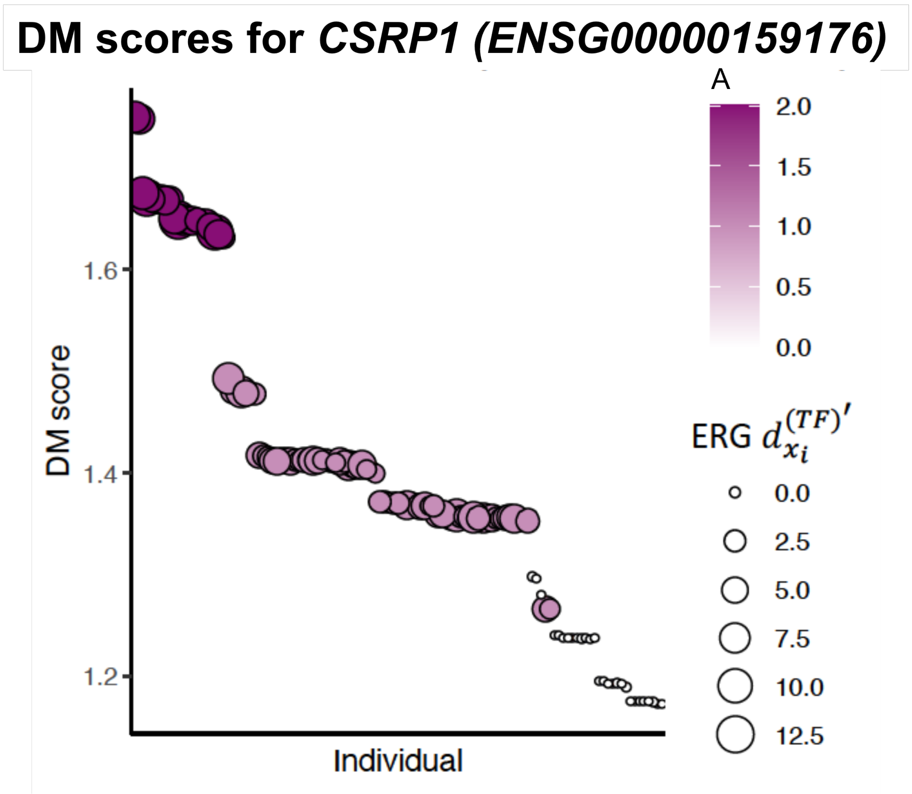

Figure S22: **DM scores of *CSRP1* for 119 individuals in Yoruba population.** Point color intensity corresponds to the individual's alternate allele dosage A for the SNP within the ERG binding motif, and point size corresponds to the TF disruption $d_{x_i}^{(TF)'}$ score of ERG.

Supplementary Tables

Table S1: Inputs to EGRET.

Name	Symbol	Source	Notes
Reference motif prior	M	FIMO (Grant et al., 2011) motif calls	TF motif locations called on a reference genome.
eQTLs	β	GTEx (Lonsdale et al., 2013)	eQTLs from a public database can be used, it is not necessary for eQTLs to be determined in the investigator's specific study population.
SNPs	s	Individual(s) genotype(s)	Genotypes of the specific individuals for which the investigator wishes to construct EGRET networks for. These are the variants used to tailor the networks to a specific individual.
Individual(s)	x	Investigator's study individual/population	This is the individual(s) for which genotype-specific EGRET networks will be constructed.
QBiC predictions	q	QBiC (Martin et al., 2019)	QBiC must be applied to the SNPs s from the individual(s) x which overlap with eQTLs within promoter-residing motifs.
PPI	P	StringDB (Mering et al., 2003)	Protein-protein interactions from a public database.
Gene expression	C	GTEx (Lonsdale et al., 2013)	RNA-seq measurements across a population are needed to estimate co-expression relationships. C does not need to be estimated from individuals for which specific networks are being constructed, it can be derived from databases such as GTEx.

Table S2: **Thresholds:** Different thresholds/thresholding strategies used during EGRET network construction and analysis.

Description	Metric	Threshold
QBiC effects	p-value	1×10^{-4} for models trained on human PBMs; 1×10^{-20} models trained on non-human PBMs
GTEx eQTLs	p-value	Empirical p-value of the gene closest to the 0.05 FDR
Banovich et al. eQTLs	p-value	1×10^{-5}
Motifs	p-value	1×10^{-4}
Edge disruption scores	$d^{(E)}_{x_{ij}}$	0.35 (see Note S6.2)
Allele-specific expression variants	FDR	0.1
caQTL variants	FDR	0.1
TF disruption scores	$d^{(TF)}_{x_i}$	Define top 10% of TF disruption scores as "high." These are used as input in Fisher's exact test enrichment analysis.
ALPACA DM scores	DM_j	No thresholding; genes were ranked by DM score prior to GO enrichment using GOrilla which requires only a ranked list.
ALPACA DM scores for CAD genes	DM_j	CAD-gene DM scores in CMs within the top 10% are considered "high".
ALPACA DM scores for CD genes	DM_j	CD-gene DM scores in LCLs within the top 10% are considered "high".
Regulatory difference score (genes)	R_j	Define top 10% of regulatory difference scores as "high." These are used as input in Fisher's exact test enrichment analysis against ASE and caQTL variants.
Regulatory difference score (edges)	R_{ij}	Define top 10% of regulatory difference scores for edges as "high." These are used as input in Fisher's exact test enrichment analysis against the differential ChIP-seq network.

Table S3: **Computational requirements:** Statistics on computational requirements for running EGRET.

Metric	Pre-processing	EGRET
Memory peak (Gb)	25.848184	77.884748
User time (s)	8742.26	19753.61
System time (s)	112.93	7355.42
Wall time (h:mm:ss)	1:18:52	1:14:42

Table S4: Calculated outputs from EGRET.

Name	Symbol	Source/Formula	Notes
Egret prior	E	$M - \sum_s \lvert q_{s_{ij}} A_{s_{ij}} \beta_{s_{ij}} \rvert$	EGRET prior E is the genotype-edited form of M and is combined with C and P during message passing.
EGRET GRN	E^*	Message passing	Genotype-specific EGRET GRN E^* produced by message passing of E, C, and P. A high edge weight indicates a putative regulatory relationship where as a low weight indicates lack of a regulatory relationship.
Baseline GRN	B^*	Message passing	Baseline GRN produced by message passing of M, C, and P. A high edge weight indicates a putative regulatory relationship where as a low weight indicates lack of a regulatory relationship.
Edge disruption score	$d_{x_{ij}}^{(E)}$	$d_{x_{ij}}^{(E)} = \lvert E_{x_{ij}}^* - B_{ij}^* \rvert$	Edge disruption scores measure the extent to which a particular TF-gene regulatory relationship is disrupted by genetic variants in a given individual. A high value of $d_{x_{ij}}^{(E)}$ indicates that the regulatory relationship between TF i and gene j is likely disrupted by genetic variants.
TF disruption score	$d_{x_i}^{(TF)}$	$d_{x_i}^{(TF)} = \sum_j \left\lvert E_{x_{ij}}^* - B_{ij}^* \right\rvert$	TF disruption scores measure the extent to which a particular TF's binding sites in promoters across the genome are disrupted by genetic variants in a given individual. A high value of $d_{x_i}^{(TF)}$ indicates that the binding sites of TF i are likely disrupted by genetic variants.
Gene disruption score	$d_{x_j}^{(G)}$	$d_{x_j}^{(G)} = \sum_i \left\lvert E_{x_{ij}}^* - B_{ij}^* \right\rvert$	Gene disruption scores measure extent to which a particular gene's promoter region is disrupted by genetic variants in a given individual. A high value of $d_{x_j}^{(G)}$ indicates that the promoter region of gene j is likely disrupted by genetic variants.
Edge regulatory difference score	$R_{ij}^{(E)}$	$R_{ij}^{(E)} = \left\lvert d_{g_{ij}}^{(E)} - d_{k_{ij}}^{(E)} \right\rvert$	Edge regulatory difference scores compare the edge disruption scores between two individuals, and thus measure the extent to which a TF-gene relationship is differentially disrupted by genetic variants between two individuals.

Gene regulatory difference score	$R_j^{(G)}$	$R_j^{(G)} = \sum_i R_{ij}^{(E)}$	Gene regulatory difference scores sum the edge regulatory difference scores per gene, and thus measure the extent to which the promoter region of a gene is differentially disrupted by genetic variants when comparing two individuals.
Differential modularity score	DM	ALPACA	ALPACA, when applied to compare an EGRET GRN E^* with a baseline B^*, calculates a differential modularity score for each gene. The DM score indicates the contribution of that gene to the differential modularity between the baseline and EGRET GRNs.

Table S5: Improvement in AUC-ROC for the prediction of the ChIP-seq regulatory network in GM12878 when using EGRET edge weights, over using baseline network edge-weights, for different cutoffs of $d_{x_{ij}}^{(E)}$. Total number of negatives (N), total number of positives (P), improvement in the AUC-ROC as well as the Delong p-value for the improvement are reported.

$d_{x_{ij}}^{(E)}$ **cutoff**	**N**	**P**	**AUC improvement**	**Delong p-value**
0.1	226	133	-0.05	0.96
0.15	132	81	-0.07	0.95
0.2	90	76	-0.01	0.55
0.25	72	75	0.08	0.07
0.3	70	72	0.09	0.05
**0.35**	**57**	**65**	**0.14**	**0.01**
0.4	57	64	0.13	0.02
0.45	57	64	0.13	0.02
0.5	57	64	0.13	0.02
0.55	57	62	0.11	0.04
0.6	57	62	0.11	0.04
0.65	57	61	0.10	0.05
0.7	57	61	0.10	0.05
0.75	57	58	0.07	0.12
0.8	57	58	0.07	0.12
0.85	57	58	0.07	0.12
0.9	57	57	0.06	0.16
1	56	56	0.06	0.16

Table S6: Improvement in AUC-ROC for the prediction of the ChIP-seq regulatory network in K562 when using EGRET edge weights, over using baseline network edge-weights, for different cutoffs of $d^{(E)}_{x_{ij}}$. Total number of negatives (N), total number of positives (P), improvement in the AUC-ROC as well as the Delong p-value for the improvement are reported.

$d^{(E)}_{x_{ij}}$ **cutoff**	**N**	**P**	**AUC improvement**	**Delong p-value**
0.1	750	547	-0.01	0.88
0.15	408	283	-0.03	0.90
0.2	235	161	-0.05	0.93
0.25	149	127	-0.01	0.55
0.3	105	97	0.03	0.29
**0.35**	**75**	**78**	**0.11**	**0.03**
0.4	68	72	0.14	0.01
0.45	67	70	0.13	0.02
0.5	67	69	0.13	0.02
0.55	67	68	0.12	0.03
0.6	67	68	0.12	0.03
0.65	64	63	0.12	0.03
0.7	61	57	0.11	0.05
0.75	61	57	0.11	0.05
0.8	61	57	0.11	0.05
0.85	61	57	0.11	0.05
0.9	61	57	0.11	0.05
1	61	56	0.11	0.05

Table S7: GWAS catalog study references for CAD genes.

PMID	Study	Ref
21239051	Identification of ADAMTS7 as a novel locus for coronary atherosclerosis and association of ABO with myocardial infarction in the presence of coronary atherosclerosis: two genome-wide association studies.	(Reilly et al., 2011)
24262325	Shared genetic susceptibility to ischemic stroke and coronary artery disease: a genome-wide analysis of common variants.	(Dichgans et al., 2014)
26343387	A comprehensive 1,000 Genomes-based genome-wide association meta-analysis of coronary artery disease.	(Nikpay et al., 2015)
26708285	A genome-wide association study reveals susceptibility loci for myocardial infarction/coronary artery disease in Saudi Arabs.	(Wakil et al., 2016)
28714974	Genetic analysis in UK Biobank links insulin resistance and transendothelial migration pathways to coronary artery disease.	(Klarin et al., 2017)
29212778	Identification of 64 Novel Genetic Loci Provides an Expanded View on the Genetic Architecture of Coronary Artery Disease.	(van der Harst and Verweij, 2018)
29263402	Genome-wide association study identifies a missense variant at APOA5 for coronary artery disease in Multi-Ethnic Cohorts from Southeast Asia.	(Han et al., 2017)
29472232	Genome-Wide Association and Functional Studies Identify SCML4 and THSD7A as Novel Susceptibility Genes for Coronary Artery Disease.	(Li et al., 2018)
30104761	Efficiently controlling for case-control imbalance and sample relatedness in large-scale genetic association studies.	(Zhou et al., 2018)
30402224	Identification of 26 novel loci that confer susceptibility to early-onset coronary artery disease in a Japanese population.	(Yamada et al., 2018)

Table S8: GWAS catalog study references for CD genes.

PMID	Study	Ref
17435756	Genome-wide association study identifies new susceptibility loci for Crohn disease and implicates autophagy in disease pathogenesis.	(Rioux et al., 2007)
17447842	Novel Crohn disease locus identified by genome-wide association maps to a gene desert on 5p13.1 and modulates expression of PTGER4.	(Libioulle et al., 2007)
17554261	Sequence variants in the autophagy gene IRGM and multiple other replicating loci contribute to Crohn's disease susceptibility.	(Parkes et al., 2007)
17554300	Genome-wide association study of 14,000 cases of seven common diseases and 3,000 shared controls.	(Consortium et al., 2007)
17684544	Systematic association mapping identifies NELL1 as a novel IBD disease gene.	(Franke et al., 2007)
17804789	Genome-wide association study for Crohn's disease in the Quebec Founder Population identifies multiple validated disease loci.	(Raelson et al., 2007)
18587394	Genome-wide association defines more than 30 distinct susceptibility loci for Crohn's disease.	(Barrett et al., 2008)
20570966	Fucosyltransferase 2 (FUT2) non-secretor status is associated with Crohn's disease.	(McGovern et al., 2010)
21102463	Genome-wide meta-analysis increases to 71 the number of confirmed Crohn's disease susceptibility loci.	(Franke et al., 2010)
22293688	1000 Genomes-based imputation identifies novel and refined associations for the Wellcome Trust Case Control Consortium phase 1 Data.	(Huang et al., 2012)
22412388	A genome-wide scan of Ashkenazi Jewish Crohn's disease suggests novel susceptibility loci.	(Kenny et al., 2012)
22936669	A genome-wide association study on a southern European population identifies a new Crohn's disease susceptibility locus at RBX1-EP300.	(Julià et al., 2013)
23128233	Host-microbe interactions have shaped the genetic architecture of inflammatory bowel disease.	(Jostins et al., 2012)
23266558	A genome-wide association study identifies 2 susceptibility Loci for Crohn's disease in a Japanese population.	(Yamazaki et al., 2013)
23850713	Genome-wide association study of Crohn's disease in Koreans revealed three new susceptibility loci and common attributes of genetic susceptibility across ethnic populations.	(Yang et al., 2014)
25489960	Immunochip analysis identification of 6 additional susceptibility loci for Crohn's disease in Koreans.	(Yang et al., 2015)
26192919	Association analyses identify 38 susceptibility loci for inflammatory bowel disease and highlight shared genetic risk across populations.	(Liu et al., 2015)
26278503	Characterization of genetic loci that affect susceptibility to inflammatory bowel diseases in African Americans.	(Huang et al., 2015)
26891255	HLA-C*01 is a Risk Factor for Crohn's Disease.	(Jung et al., 2016)
28008999	Genetic architecture differences between pediatric and adult-onset inflammatory bowel diseases in the Polish population.	(Ostrowski et al., 2016)
28067908	Genome-wide association study implicates immune activation of multiple integrin genes in inflammatory bowel disease.	(De Lange et al., 2017)
30500874	A genome-wide association study identifying RAP1A as a novel susceptibility gene for Crohn's disease in Japanese individuals.	(Kakuta et al., 2019)

Supplementary Table Attachments

Table S9: See supplementary file attachment Supplementary_Table_S9.txt

Table S10: See supplementary file attachment Supplementary_Table_S10.txt

Table S11: See supplementary file attachment Supplementary_Table_S11.txt

References

Banovich NE, Li YI, Raj A, Ward MC, Greenside P, Calderon D, Tung PY, Burnett JE, Myrthil M, Thomas SM, et al.. 2018. Impact of regulatory variation across human ipscs and differentiated cells. *Genome research* **28**: 122–131.

Barrett JC, Hansoul S, Nicolae DL, Cho JH, Duerr RH, Rioux JD, Brant SR, Silverberg MS, Taylor KD, Barmada MM, et al.. 2008. Genome-wide association defines more than 30 distinct susceptibility loci for crohn's disease. *Nature genetics* **40**: 955–962.

Bolstad BM, Irizarry RA, Åstrand M, and Speed TP. 2003. A comparison of normalization methods for high density oligonucleotide array data based on variance and bias. *Bioinformatics* **19**: 185–193.

Boughton AP, Welch RP, Flickinger M, VandeHaar P, Taliun D, Abecasis GR, and Boehnke M. 2021. LocusZoom.js: interactive and embeddable visualization of genetic association study results. *Bioinformatics* Btab186.

Buniello A, MacArthur JAL, Cerezo M, Harris LW, Hayhurst J, Malangone C, McMahon A, Morales J, Mountjoy E, Sollis E, et al.. 2019. The nhgri-ebi gwas catalog of published genome-wide association studies, targeted arrays and summary statistics 2019. *Nucleic acids research* **47**: D1005–D1012.

Chèneby J, Gheorghe M, Artufel M, Mathelier A, and Ballester B. 2018. Remap 2018: an updated atlas of regulatory regions from an integrative analysis of dna-binding chip-seq experiments. *Nucleic acids research* **46**: D267–D275.

Consortium WTCC et al.. 2007. Genome-wide association study of 14,000 cases of seven common diseases and 3,000 shared controls. *Nature* **447**: 661.

De Lange KM, Moutsianas L, Lee JC, Lamb CA, Luo Y, Kennedy NA, Jostins L, Rice DL, Gutierrez-Achury J, Ji SG, et al.. 2017. Genome-wide association study implicates immune activation of multiple integrin genes in inflammatory bowel disease. *Nature genetics* **49**: 256–261.

Degner JF, Pai AA, Pique-Regi R, Veyrieras JB, Gaffney DJ, Pickrell JK, De Leon S, Michelini K, Lewellen N, Crawford GE, et al.. 2012. Dnase i sensitivity qtls are a major determinant of human expression variation. *Nature* **482**: 390–394.

Dichgans M, Malik R, König IR, Rosand J, Clarke R, Gretarsdottir S, Thorleifsson G, Mitchell BD, Assimes TL, Levi C, et al.. 2014. Shared genetic susceptibility to ischemic stroke and coronary artery disease: a genome-wide analysis of common variants. *Stroke* **45**: 24–36.

Eden E, Navon R, Steinfeld I, Lipson D, and Yakhini Z. 2009. Gorilla: a tool for discovery and visualization of enriched go terms in ranked gene lists. *BMC bioinformatics* **10**: 1–7.

Franke A, Hampe J, Rosenstiel P, Becker C, Wagner F, Häsler R, Little RD, Huse K, Ruether A, Balschun T, et al.. 2007. Systematic association mapping identifies nell1 as a novel ibd disease gene. *PloS one* **2**: e691.

Franke A, McGovern DP, Barrett JC, Wang K, Radford-Smith GL, Ahmad T, Lees CW, Balschun T, Lee J, Roberts R, et al.. 2010. Genome-wide meta-analysis increases to 71 the number of confirmed crohn's disease susceptibility loci. *Nature genetics* **42**: 1118–1125.

Glass K, Huttenhower C, Quackenbush J, and Yuan GC. 2013. Passing messages between biological networks to refine predicted interactions. *PloS one* **8**: e64832.

Glass K, Quackenbush J, Spentzos D, Haibe-Kains B, and Yuan GC. 2015. A network model for angiogenesis in ovarian cancer. *BMC Bioinformatics* **16**: 115.

Grant CE, Bailey TL, and Noble WS. 2011. FIMO: scanning for occurrences of a given motif. *Bioinformatics* **27**: 1017–1018.

GTEx Consortium. 2017. Genetic effects on gene expression across human tissues. *Nature* **550**: 204–213.

Han Y, Dorajoo R, Chang X, Wang L, Khor CC, Sim X, Cheng CY, Shi Y, Tham YC, Zhao W, et al.. 2017. Genome-wide association study identifies a missense variant at apoa5 for coronary artery disease in multi-ethnic cohorts from southeast asia. *Scientific reports* **7**: 1–11.

van der Harst P and Verweij N. 2018. Identification of 64 novel genetic loci provides an expanded view on the genetic architecture of coronary artery disease. *Circulation research* **122**: 433–443.

Huang C, Haritunians T, Okou DT, Cutler DJ, Zwick ME, Taylor KD, Datta LW, Maranville JC, Liu Z, Ellis S, et al.. 2015. Characterization of genetic loci that affect susceptibility to inflammatory bowel diseases in african americans. *Gastroenterology* **149**: 1575–1586.

Huang J, Ellinghaus D, Franke A, Howie B, and Li Y. 2012. 1000 genomes-based imputation identifies novel and refined associations for the wellcome trust case control consortium phase 1 data. *European Journal of Human Genetics* **20**: 801–805.

Jostins L, Ripke S, Weersma RK, Duerr RH, McGovern DP, Hui KY, Lee JC, Schumm LP, Sharma Y, Anderson CA, et al.. 2012. Host–microbe interactions have shaped the genetic architecture of inflammatory bowel disease. *Nature* **491**: 119–124.

Julià A, Domènech E, Ricart E, Tortosa R, García-Sánchez V, Gisbert JP, Mateu PN, Gutiérrez A, Gomollón F, Mendoza JL, et al.. 2013. A genome-wide association study on a southern european population identifies a new crohn's disease susceptibility locus at rbx1-ep300. *Gut* **62**: 1440–1445.

Jung ES, Cheon JH, Lee JH, Park SJ, Jang HW, Chung SH, Park MH, Kim TG, Oh HB, Yang SK, et al.. 2016. Hla-c* 01 is a risk factor for crohn's disease. *Inflammatory bowel diseases* **22**: 796–806.

Kakuta Y, Kawai Y, Naito T, Hirano A, Umeno J, Fuyuno Y, Liu Z, Li D, Nakano T, Izumiyama Y, et al.. 2019. A genome-wide association study identifying rap1a as a novel susceptibility gene for crohn's disease in japanese individuals. *Journal of Crohn's and Colitis* **13**: 648–658.

Kalita CA, Brown CD, Freiman A, Isherwood J, Wen X, Pique-Regi R, and Luca F. 2018. High-throughput characterization of genetic effects on dna–protein binding and gene transcription. *Genome research* **28**: 1701–1708.

Kenny EE, Pe'er I, Karban A, Ozelius L, Mitchell AA, Ng SM, Erazo M, Ostrer H, Abraham C, Abreu MT, et al.. 2012. A genome-wide scan of ashkenazi jewish crohn's disease suggests novel susceptibility loci. *PLoS Genet* **8**: e1002559.

Klarin D, Zhu QM, Emdin CA, Chaffin M, Horner S, McMillan BJ, Leed A, Weale ME, Spencer CC, Aguet F, et al.. 2017. Genetic analysis in uk biobank links insulin resistance and transendothelial migration pathways to coronary artery disease. *Nature genetics* **49**: 1392.

Lawrence M, Huber W, Pagès H, Aboyoun P, Carlson M, Gentleman R, Morgan M, and Carey V. 2013. Software for computing and annotating genomic ranges. *PLoS Computational Biology* **9**.

Li Y, Wang DW, Chen Y, Chen C, Guo J, Zhang S, Sun Z, Ding H, Yao Y, Zhou L, et al.. 2018. Genome-wide association and functional studies identify scml4 and thsd7a as novel susceptibility genes for coronary artery disease. *Arteriosclerosis, thrombosis, and vascular biology* **38**: 964–975.

Li YI, Van De Geijn B, Raj A, Knowles DA, Petti AA, Golan D, Gilad Y, and Pritchard JK. 2016. Rna splicing is a primary link between genetic variation and disease. *Science* **352**: 600–604.

Libioulle C, Louis E, Hansoul S, Sandor C, Farnir F, Franchimont D, Vermeire S, Dewit O, De Vos M, Dixon A, et al.. 2007. Novel crohn disease locus identified by genome-wide association maps to a gene desert on 5p13. 1 and modulates expression of ptger4. *PLoS Genet* **3**: e58.

Liu JZ, Van Sommeren S, Huang H, Ng SC, Alberts R, Takahashi A, Ripke S, Lee JC, Jostins L, Shah T, et al.. 2015. Association analyses identify 38 susceptibility loci for inflammatory bowel disease and highlight shared genetic risk across populations. *Nature genetics* **47**: 979–986.

Lonsdale J, Thomas J, Salvatore M, Phillips R, Lo E, Shad S, Hasz R, Walters G, Garcia F, Young N, et al.. 2013. The genotype-tissue expression (GTEx) project. *Nature genetics* **45**: 580.

Martin V, Zhao J, Afek A, Mielko Z, and Gordân R. 2019. Qbic-pred: quantitative predictions of transcription factor binding changes due to sequence variants. *Nucleic acids research* **47**: W127–W135.

McGovern DP, Jones MR, Taylor KD, Marciante K, Yan X, Dubinsky M, Ippoliti A, Vasiliauskas E, Berel D, Derkowski C, et al.. 2010. Fucosyltransferase 2 (fut2) non-secretor status is associated with crohn's disease. *Human molecular genetics* **19**: 3468–3476.

Mering Cv, Huynen M, Jaeggi D, Schmidt S, Bork P, and Snel B. 2003. String: a database of predicted functional associations between proteins. *Nucleic acids research* **31**: 258–261.

Nikpay M, Goel A, Won HH, Hall LM, Willenborg C, Kanoni S, Saleheen D, Kyriakou T, Nelson CP, Hopewell JC, et al.. 2015. A comprehensive 1000 genomes–based genome-wide association meta-analysis of coronary artery disease. *Nature genetics* **47**: 1121.

Ongen H, Buil A, Brown AA, Dermitzakis ET, and Delaneau O. 2016. Fast and efficient qtl mapper for thousands of molecular phenotypes. *Bioinformatics* **32**: 1479–1485.

Ostrowski J, Paziewska A, Lazowska I, Ambrozkiewicz F, Goryca K, Kulecka M, Rawa T, Karczmarski J, Dabrowska M, Zeber-Lubecka N, et al.. 2016. Genetic architecture differences between pediatric and adult-onset inflammatory bowel diseases in the polish population. *Scientific reports* **6**: 39831.

Padi M and Quackenbush J. 2018. Detecting phenotype-driven transitions in regulatory network structure. *NPJ systems biology and applications* **4**: 1–12.

Parkes M, Barrett JC, Prescott NJ, Tremelling M, Anderson CA, Fisher SA, Roberts RG, Nimmo ER, Cummings FR, Soars D, et al.. 2007. Sequence variants in the autophagy gene irgm and multiple other replicating loci contribute to crohn's disease susceptibility. *Nature genetics* **39**: 830–832.

Raelson JV, Little RD, Ruether A, Fournier H, Paquin B, Van Eerdewegh P, Bradley W, Croteau P, Nguyen-Huu Q, Segal J, et al.. 2007. Genome-wide association study for crohn's disease in the quebec founder population identifies multiple validated disease loci. *Proceedings of the National Academy of Sciences* **104**: 14747–14752.

Raudvere U, Kolberg L, Kuzmin I, Arak T, Adler P, Peterson H, and Vilo J. 2019. g: Profiler: a web server for functional enrichment analysis and conversions of gene lists (2019 update). *Nucleic acids research* **47**: W191–W198.

Reilly MP, Li M, He J, Ferguson JF, Stylianou IM, Mehta NN, Burnett MS, Devaney JM, Knouff CW, Thompson JR, et al.. 2011. Identification of adamts7 as a novel locus for coronary atherosclerosis and association of abo with myocardial infarction in the presence of coronary atherosclerosis: two genome-wide association studies. *The Lancet* **377**: 383–392.

Rioux JD, Xavier RJ, Taylor KD, Silverberg MS, Goyette P, Huett A, Green T, Kuballa P, Barmada MM, Datta LW, et al.. 2007. Genome-wide association study identifies new susceptibility loci for crohn disease and implicates autophagy in disease pathogenesis. *Nature genetics* **39**: 596–604.

Robin X, Turck N, Hainard A, Tiberti N, Lisacek F, Sanchez JC, and Müller M. 2011. proc: an open-source package for r and s+ to analyze and compare roc curves. *BMC Bioinformatics* **12**: 77.

Saito T and Rehmsmeier M. 2017. Precrec: fast and accurate precision-recall and roc curve calculations in r. *Bioinformatics* **33** (**1**): 145–147.

Sonawane AR, Platig J, Fagny M, Chen CY, Paulson JN, Lopes-Ramos CM, DeMeo DL, Quackenbush J, Glass K, and Kuijjer ML. 2017. Understanding tissue-specific gene regulation. *Cell reports* **21**: 1077–1088.

Tange O. 2011. Gnu parallel-the command-line power tool.; login: The usenix magazine, 36 (1): 42–47.

Wakil SM, Ram R, Muiya NP, Mehta M, Andres E, Mazhar N, Baz B, Hagos S, Alshahid M, Meyer BF, et al.. 2016. A genome-wide association study reveals susceptibility loci for myocardial infarction/coronary artery disease in saudi arabs. *Atherosclerosis* **245**: 62–70.

Yamada Y, Yasukochi Y, Kato K, Oguri M, Horibe H, Fujimaki T, Takeuchi I, and Sakuma J. 2018. Identification of 26 novel loci that confer susceptibility to early-onset coronary artery disease in a japanese population. *Biomedical reports* **9**: 383–404.

Yamazaki K, Umeno J, Takahashi A, Hirano A, Johnson TA, Kumasaka N, Morizono T, Hosono N, Kawaguchi T, Takazoe M, et al.. 2013. A genome-wide association study identifies 2 susceptibility loci for crohn's disease in a japanese population. *Gastroenterology* **144**: 781–788.

Yang SK, Hong M, Choi H, Zhao W, Jung Y, Haritunians T, Ye BD, Kim KJ, Park SH, Lee I, et al.. 2015. Immunochip analysis identification of 6 additional susceptibility loci for crohn's disease in koreans. *Inflammatory bowel diseases* **21**: 1–7.

Yang SK, Hong M, Zhao W, Jung Y, Baek J, Tayebi N, Kim KM, Ye BD, Kim KJ, Park SH, et al.. 2014. Genome-wide association study of crohn's disease in koreans revealed three new susceptibility loci and common attributes of genetic susceptibility across ethnic populations. *Gut* **63**: 80–87.

Zhou B, Ho SS, Greer SU, Zhu X, Bell JM, Arthur JG, Spies N, Zhang X, Byeon S, Pattni R, et al.. 2019. Comprehensive, integrated, and phased whole-genome analysis of the primary encode cell line k562. *Genome research* **29**: 472–484.

Zhou W, Nielsen JB, Fritsche LG, Dey R, Gabrielsen ME, Wolford BN, LeFaive J, VandeHaar P, Gagliano SA, Gifford A, et al.. 2018. Efficiently controlling for case-control imbalance and sample relatedness in large-scale genetic association studies. *Nature genetics* **50**: 1335–1341.
